# Photo‐Assisted Rechargeable Metal Batteries: Principles, Progress, and Perspectives

**DOI:** 10.1002/advs.202402448

**Published:** 2024-06-14

**Authors:** Pengpeng Zhang, Meng Cai, Yixin Wei, Jingbo Zhang, Kaizhen Li, Sembukuttiarachilage Ravi Pradip Silva, Guosheng Shao, Peng Zhang

**Affiliations:** ^1^ School of Materials Science and Engineering Zhengzhou University Zhengzhou 450001 China; ^2^ State Centre for International Cooperation on Designer Low‐Carbon & Environmental Materials (CDLCEM) Zhengzhou University 100 Kexue Avenue Zhengzhou 450001 China; ^3^ Zhengzhou Materials Genome Institute (ZMGI) Zhengzhou 450001 China; ^4^ Nanoelectronics Center Advanced Technology Institute University of Surrey Guildford GU2 7XH UK

**Keywords:** energy conversion and storage, metal batteries, photo‐assisted rechargeable batteries, photo‐electrochemistry, Solar energy

## Abstract

The utilization of diverse energy storage devices is imperative in the contemporary society. Taking advantage of solar power, a significant environmentally friendly and sustainable energy resource, holds great appeal for future storage of energy because it can solve the dilemma of fossil energy depletion and the resulting environmental problems once and for all. Recently, photo‐assisted energy storage devices, especially photo‐assisted rechargeable metal batteries, are rapidly developed owing to the ability to efficiently convert and store solar energy and the simple configuration, as well as the fact that conventional Li/Zn‐ion batteries are widely commercialized. Considering many puzzles arising from the rapid development of photo‐assisted rechargeable metal batteries, this review commences by introducing the fundamental concepts of batteries and photo‐electrochemistry, followed by an exploration of the current advancements in photo‐assisted rechargeable metal batteries. Specifically, it delves into the elucidation of device components, operating principles, types, and practical applications. Furthermore, this paper categorizes, specifies, and summarizes several detailed examples of photo‐assisted energy storage devices. Lastly, it addresses the challenges and bottlenecks faced by these energy storage systems while providing future perspectives to facilitate their transition from laboratory research to industrial implementation.

## Graphical abstract

This review begins with the concepts of batteries and photo‐electrochemistry and proceeds to the current state of the art of photo‐assisted rechargeable metal batteries, where the device components, operating principles, and applications are specifically elucidated. Lastly, it addresses the challenges faced by these energy storage systems while providing perspectives to facilitate their transition from laboratory to industrial implementation.

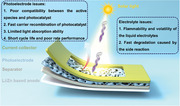

## 1 Introduction

The rapid development of science, technology, and industry has led to a frantic consumption of traditional fossil energy sources. Therefore, there has been a shift in focus toward the efficient utilization of green, sustainable power sources like solar, wind, and nuclear to replace traditional oil and coal. In specific, the sun provides approximately four million joules (1 EJ = 10^18^ J) of solar energy to our planet annually, with roughly 5 × 10^4^ EJ being easily accessible.^[^ ^1^ ^]^ Based on the fact that people can get all the energy they need from sunlight, a great deal of effort has been poured into the study of converting solar energy into electrical energy for numerous applications and devices with the help of photovoltaic devices.^[^ ^2^, ^3^ ^]^ Solar cells are known to effectively convert solar energy into electricity. However, it has major drawbacks due to the unsustainable and intermittent nature of photo energy, which can result in a waste of the photovoltaic conversion energy.

**Figure 1:**
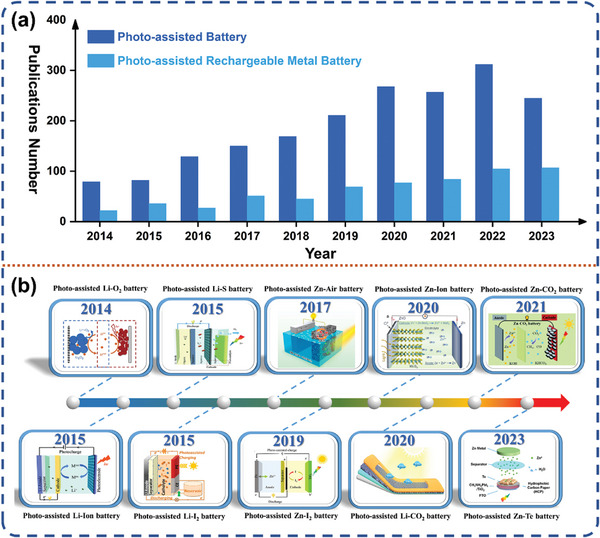
a) Statistics of publications based on Photo‐assisted battery from 1 January 2014 to 1 July 2023 by searching “Photo/Light/optical‐assisted/field/involved” and “Battery/Rechargeable Metal Battery” as “topic” in the website: Web of Science. b) Timeline of major developments in Photo‐assisted rechargeable metal battery in the past decade. Reproduced with permission.^[^ ^21^ ^]^ Copyright 2014, Springer Nature. Reproduced with permission.^[^ ^22^ ^]^ Copyright 2015, Royal Society of Chemistry. Reproduced with permission.^[^ ^23^ ^]^ Copyright 2015, Wiley‐VCH. Reproduced with permission.^[^ ^102^ ^]^ Copyright 2015, American Chemical Society. Reproduced with permission.^[^ ^7^ ^]^ Copyright 2017, Elsevier Ltd. Reproduced with permission.^[^ ^127^ ^]^ Copyright 2019, Wiley‐VCH. Reproduced with permission.^[^ ^125^ ^]^ Copyright 2020, Royal Society of Chemistry. Reproduced with permission.^[^ ^93^ ^]^ Copyright 2020, Wiley‐VCH. Reproduced with permission.^[^ ^128^ ^]^ Copyright 2021, Royal Society of Chemistry. Reproduced with permission.^[^ ^129^ ^]^ Copyright 2023, American Chemical Society.

To address this challenge and achieve efficient utilization of solar energy, diverse solar photovoltaic systems have been integrated with other electrochemical energy storage systems, such as Li metal batteries,^[^ ^4^, ^5^, ^6^ ^]^ Zn metal batteries,^[^ ^7^, ^8^, ^9^, ^10^ ^]^ Na metal batteries,^[^ ^11^ ^]^ and fuel cells.^[^ ^12^ ^]^ **Figure** **1**a shows the number of publications in the field of photo‐assisted rechargeable devices over the last decade, indicating that the research on photo‐assisted rechargeable batteries has been increasingly popular in recent years. Among many energy devices, rechargeable metal batteries have demonstrated their exceptional performance as an integrated energy storage solution. Specifically, lithium‐ion (Li‐ion) batteries are widely utilized in various industries such as electronics, transportation (including electric vehicles), and numerous other sectors.^[^ ^13^ ^]^ Zinc‐ion (Zn‐ion) batteries have also gained widespread attention due to the lower cost, non‐toxicity, safe aqueous electrolyte, and non‐flammability.^[^ ^14^, ^15^, ^16^, ^17^, ^18^ ^]^ Lithium‐oxygen (Li‐O_2_) and Zn‐air batteries based on conversion reactions have ultra‐high energy density and are considered prominent alternatives to commercial Li‐ion batteries.^[^ ^19^, ^20^ ^]^ Efficient and sustainable utilization of solar energy over large areas is achievable once it is captured and stored in rechargeable batteries. Photo‐involved energy storage devices are also beneficial for reducing the input energy and increasing the output energy of normal rechargeable metal batteries.

Early photo‐assisted charging strategy typically required external circuitry to connect separate solar photovoltaic devices with the storage battery for electricity storage. However, this method often resulted in complex device structures, redundant photoelectric conversion modes, and additional components. Naturally, it will cause the energy loss, reduced energy conversion/storage efficiency as well as increased operating costs and size/weight. In 2014, Wu et al. reported for the first time a three‐electrode system photo‐assisted Li‐O_2_ battery with an integrated light conversion/energy storage component, which effectively reduced the charging voltage of the battery under light illumination.^[^ ^21^ ^]^ This pioneering study provides an excellent research strategy and technical pathway to motivate researchers to develop a variety of integrated photo‐assisted rechargeable metal batteries to solve this challenge. In the following time, Li‐ion batteries,^[^ ^22^ ^]^ Li–S batteries,^[^ ^23^ ^]^ Zn‐air batteries,^[^ ^7^, ^24^ ^]^ Zn‐ion batteries,^[^ ^25^, ^26^ ^]^ and other energy storage systems have introduced photo‐assisted strategy, which has greatly expanded the research field of photo‐promoted charging and discharging mechanisms, and offered extensive perspectives to facilitate the progress of photo‐assisted rechargeable metal batteries, aiming for enhanced efficiency. In the chronological progression of the advancement in photo‐assisted rechargeable metal batteries, as documented by historical records (Figure 1b), one can see a gradual transition in the configuration of these devices from a three‐electrode system to a simpler, more practical two‐electrode configuration. With the deepening of the research, a deeper and deeper understanding of the photoelectric reaction inside the photocathode has been gained. Photo‐assisted batteries can augment the electrochemical capability of rechargeable batteries and provide a novel approach for solar energy storage. Different from conventional energy storage devices, photo‐assisted batteries convert solar energy into electrical energy directly and store it as chemical energy. While significant advances have been achieved, there are still many topics that need to be addressed.

In this review, battery and photo‐electrochemical concepts as well as the working mechanism of photo‐assisted rechargeable batteries are first introduced in turn. Subsequently, this review summarizes and analyzes the progress of photoelectrode research and design strategies for photo‐assisted rechargeable metal batteries. The analysis is conducted from the perspective of battery type and includes the latest examples. Finally, the key issues and perspectives on which photo‐assisted rechargeable metal batteries should focus and research in the future are discussed, and are expected to pave the way from laboratory to industry.

## 2 Configuration and Working Mechanisms

In this section, the concept of rechargeable secondary battery will be first introduced. Subsequently, a comprehensive analysis of the photoelectrochemical mechanism will be presented. Lastly, a concise overview will be provided on the utilization of the photoelectric effect in facilitating photo‐assisted rechargeable metal batteries.

### 2.1 Battery Concept

A rechargeable secondary battery typically comprises a cathode, separator, electrolyte and anode. There are two distinct categories of reactions observed in rechargeable secondary batteries: intercalation and conversion mechanisms. For instance, Li‐ion batteries based on intercalation reaction chemistry are highly commercialized rechargeable batteries today. The storage and release of electrical energy in Li‐ion batteries is dependent on the intercalation and deintercalation of Li ions within the cathode and anode layers.^[^ ^27^ ^]^ Other types of Li‐ion and lithium‐metal batteries, such as Li–S and Li‐O_2_ batteries which adopt the Li metal as the anode are based on conversion reactions to supply power to an external load. For instance, Li–S batteries facilitate the conversion of chemical energy into electrical energy and vice versa by means of a redox reaction involving Li metal and elemental sulfur. This reaction involves the gain and loss of electrons.^[^ ^28^ ^]^ During the charging process, Li ions are reduced to Li metal, which is then oxidized back to Li ions during discharge. Similarly, sulfur gains electrons and is reduced to Li_2_S during the discharging process, while loses electrons and is regenerated as a sulfur monomer during the charging process. When the battery undergoes charging, an external energy supply is linked to it, leading to the flow of a charging current that facilitates a reversible electrochemical process between the cathode and anode inside the battery. It converts the electrical energy into chemical energy, which is then stored in the battery. When the battery is depleted, the chemical energy that was stored within it undergoes a reversal of electrochemical reaction, resulting in the conversion back into electrical energy.

### 2.2 Photo‐Electrochemistry Concept

Photo‐electrochemistry, as the name implies, is a photo‐driven electrochemical reaction involving light trapping, excitation of electrons, separation and migration of photo‐generated carriers and ultimately redox reaction. Using a single semiconductor as an example, photoelectrons are generated upon excitation by incident light with an energy that matches or exceeds the bandgap energy *E_g_* (also referred to as the forbidden bandwidth). These photoelectrons have the ability to leap from the valence band (VB) of a semiconductor to the conduction band (CB), resulting in holes within the VB. The reduction reaction involves the photoelectrons, while the oxidation reaction requires the addition of holes. The semiconductor's ability to absorb light and engage in redox reactions is determined by the forbidden bandwidth and CB/VB position, respectively. Generally, a smaller bandgap leads to better conductivity in the semiconductor and a broader light absorption range. It is well‐known that as we move from the ultraviolet to the infrared region, the wavelength increases and the frequency decreases, with the energy of light becoming lower and lower. A smaller bandgap means the energy barrier for photo‐generated electrons in semiconductor need to overcome is lower, allowing the light response range can to expand into the visible and even infrared light regions, thereby making better use of solar energy. On the other hand, for a semiconductor to successfully catalyze a redox reaction, its CB position needs to be higher than the reduction potential of the redox reaction, and its VB position needs to be lower than the oxidation potential. In this way, the photo‐generated electrons in the CB of semiconductor will have enough reduction ability to participate in the reduction reaction. Correspondingly, the photo‐generated holes in the VB can effectively oxidize the reduction product. This is also the practical principle for selecting appropriate photoelectrode materials for photo‐assisted energy storage devices. Furthermore, the photochemical stability of semiconductor materials is a crucial factor to consider. The energy band structure determines the conductivity and the light absorption properties of the photoelectrode material and whether the photocatalyst can participate in redox reactions. Favorable photo corrosion resistance ensures the chemical stability of the photoelectrode material. Recombination of a large number of photo‐generated electron‐hole pairs occur, with only a small number of photoelectrons and photo‐generated holes utilized for the redox reaction at the active site of the photocatalyst. The most crucial factors that affect the photocatalytic performance are adequate solar energy capture and effectively isolating photo‐generated carriers. Numerous modification strategies have been proposed to enhance the light absorption capability of photocatalysts and enhance the effectiveness of carrier separation. These include doping and introducing defects to change the bandgap, as well as heterojunction and spatial separation strategies. Light‐driven electrochemical reactions have enabled various applications, including the splitting of water through photocatalysis, reduction of CO_2_, photocatalytic nitrogen fixation, and photo‐assisted rechargeable batteries.^[^ ^29^, ^30^, ^31^, ^32^ ^]^

### 2.3 Photo‐Assisted Battery Concept

**Figure 2:**
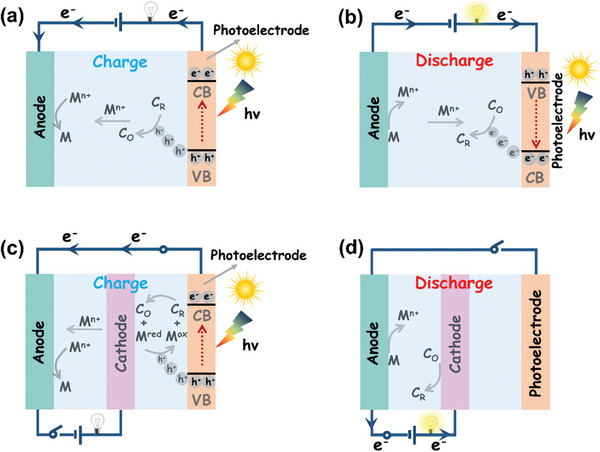
Working principle of the photo‐assisted energy storage device: a,b) The charging and discharging process of a two‐electrode device. c,d) The charging and discharging process of a three‐electrode device. Note: The direction of the blue arrow represents the flow of electrons.

A photo‐assisted rechargeable battery typically comprises two parts: one for solar energy capture and conversion, and the other for energy storage. In the early stages, photo‐assisted battery often consisted of a photovoltaic device and an energy storage battery connected by metal wires. Hence, these batteries cannot be considered as genuinely photo‐assisted battery. In the past few years, there has been a growing utilization of photosensitive and semiconductor materials in energy storage devices or their integration with electrode‐active materials to produce diverse photoelectrodes. This article focuses on photo‐assisted energy storage devices in both three‐electrode and two‐electrode configurations. Using the two‐electrode photo‐assisted system as an example, its working mechanism is shown in **Figure** **2**a,b. Throughout the charging procedure, a connection is established between the external circuit and both the photoelectrode and anode. When subjected to illumination, the photoelectrode material undergoes excitation, producing high‐energy photoelectrons that leap from the VB of the semiconductor to the CB. At the same time, positive holes are produced on the VB of the material. When the holes move to the outer layer of the active substance on the cathode side, an oxidation reaction occurs. In this reaction, the reduction product of cat‐hode (C_R_) is converted to the oxidation product (C_O_), as shown in Equation (1):

(1) $$C_{R}+h^{+}\to C_{O}$$

Simultaneously, the photoelectrons from the VB of semiconductor will travel through the external circuit to the anode side and react with the migrating metal ions M^n+^ from the cathode side, resulting in a reduction reaction that regenerate the metal M, as shown in Equation (2):

(2) $$M^{n+}+e^{-}\to M$$

Thus, the battery device realizes the transformation of electrical energy into chemical energy, which is then stored.

Throughout the discharge procedure, a connection is established between the photo‐electrode and anode to facilitate external load integration. Photoelectrons excited on the semiconductor surface react with the charging product (C_O_) of the photoelectrode in a reduction reaction, generating the discharge product (C_R_) as shown in Equation (3). Meanwhile, the photo‐holes combine with the electrons released from the oxidation reaction occurring on the anode side and then quenching, as shown in Equation (4):

(3) $$C_{O}+e^{-}\to C_{R}$$

(4) $$M\to M^{n+}+e^{-}$$

During this process, chemical energy is transformed into electrical energy and released to power the external load. In a more complex three‐electrode systems, photo‐assisted charging mode often involves a multi‐step reaction at the cathode side (Figure 2c). This reaction involves additional holes to oxidize the shuttle mediator M^red^ to M^ox^ (Equation (5): *M^red^* + *h* ^+^ → *M^ox^*) and then M^ox^ will oxidize the reduction product (C_R_) to C_O_ (Equation (6): *M^ox^* + *C_R_* → *C_O_* + *M^red^*). Unfortunately, it often leads to unavoidable energy loss and high cost. Furthermore, the conventional three‐electrode photo‐assisted configuration is limited to a single photo‐assisted charging strategy due to the limits of its structure (Figure 2d). As a result, efficient and clean solar energy cannot be utilized during the subsequent discharge stage. Therefore, this review will pay more attention to the photo‐assisted rechargeable metal batteries in the two‐electrode system. **Table** **1** shows the configurations of representative photo‐assisted rechargeable metal batteries.

**Table 1:** Working mechanisms of representative photo‐assisted rechargeable metal batteries.

Type	Anode/Cathode	Discharge	Charge	Overall
Li‐O_2_ battery	Anode	Li → Li^+^ + e^−^	Li^+^ + e^−^ → Li	Li_2_O_2_ + 2Li^+^ ↔ O_2_ + 2Li
	Cathode	O_2_ + e^−^ +2Li → Li_2_O_2_	Li_2_O_2_ + 2h^+^(M) → O_2_ + 2Li	
Li‐Ion battery	Anode	Li → Li^+^ + e^−^	Li + e^−^(M) → Li	Li_x_M_y_ ↔ Li^+^ + Li_(x‐1)_M_y_
	Cathode	Li^+^ e^−^+ Li_(x‐1)_M_y_ → Li_x_M_y_	Li_x_M_y_ + h^+^(M) → Li^+^ + M + Li_(x‐1)_M_y_	
Li–S battery	Anode	Li → Li^+^ + e^−^	Li^+^ + e^−^ → Li	S_8_ + 16 Li ↔ 8Li_2_S
	Cathode	S_8_ + 16Li^+^ +16e^−^ → 8Li_2_S	nLi_2_S + 2h^+^ → Li_2_S_n_^2−^	
			Li_2_S_n_ → n/8S_8_ + Li^+^	
Li‐CO_2_ battery	Anode	Li → Li^+^ + e^−^	Li + e^−^(M) → Li	2 Li_2_CO_3_ + C ↔ 4Li + 3CO_2_
	Cathode	4 Li^+^ + 3CO_2_ + 4e^−^ → Li_2_CO_3_ + C	Li_2_CO_3_ + C + 4h^+^(M) → 4Li^+^ + 3CO_2_	
Li‐I_2_ battery	Anode	Li → Li^+^ + e^−^	Li^+^ + e^−^(M) → Li	3I^−^ + 2 Li^+^ ↔ I_3_^−^ +2 Li
	Cathode	I_3_^−^ + 2e^−^ → 3I^−^	3I^−^ + 2h^+^(M) → I_3_^−^	
Zn‐O_2_ battery	Anode	Zn + 4OH^−^ → 2e^−^ + Zn(OH)_4_^2−^	Zn(OH)_4_^2−^ + 2e^−^(M) → Zn + 4OH^−^	2ZnO ↔ 2 Zn + O_2_
	Cathode	Zn(OH)_4_^2−^ → ZnO + H_2_O +OH^−^	4OH^−^ + 2h^+^(M) → O_2_ + 2H_2_O	
		O_2_ + 4e^−^ + 2H_2_O → 4OH^−^		
Zn‐Ion battery	Anode	Zn → Zn^2+^ + 2e^−^	Zn ^2+^ + 2e^−^(M) → Zn	Zn_x_M_y_ ↔ Zn^2+^ + Zn_(x‐1)_M_y_
	Cathode	Zn^2+^ + 2e^−^ + Zn_(x‐1)_M_y_ → Zn_x_M_y_	Zn_x_M_y_ + 2h^+^(M) → Zn^2+^ + Zn_(x‐1)_M_y_	
Zn‐I_2_ battery	Anode	Zn → Zn^2+^ + 2e^−^	Zn^2+^ + 2e^−^ → Zn	3I^−^ + Zn^2+^ ↔ I_3_^−^ + Zn
	Cathode	I_3_^−^ + 2e^−^ → 3I^−^	3I^−^ + 2h^+^ → I_3_^−^	
Al‐MnO_2_ battery	Anode	nAl → nAl^3+^ + 3ne^−^	nAl^3+^ + 3ne^−^ → nAl	MnO_2_ + nAl → Al_n_MnO_2_
	Cathode	MnO_2_ +3ne^−^ + nAl^3+^ → Al_n_MnO_2_	Al_n_MnO_2_ → MnO_2_ +3ne^−^ + nAl^3+^	
Na‐O_2_ battery	Anode	4Na → 4Na^+^ + 4e^−^	4Na^+^ + 4e^−^ → 4Na	4Na + O_2_ + 4H^+^ → 4Na^+^ + 2H_2_O
	Cathode	O_2_ + 4H^+^ + 4e^−^ → 2H_2_O	2H_2_O → O_2_ + 4H^+^ + 4e^−^	

## 3 Development and Progress

**Figure 3:**
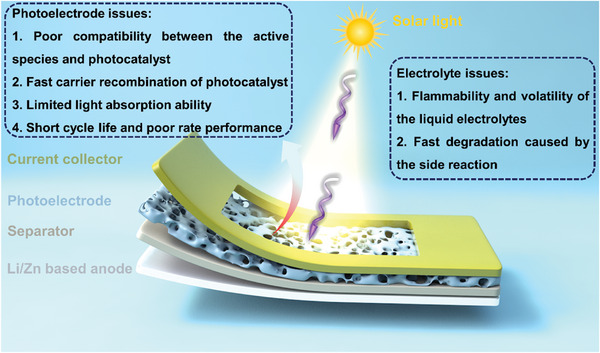
The schematic illustration of the photo‐assisted rechargeable metal batteries and the challenges.

In this section, we will discuss the historical background and recent advancements in the field of photo‐assisted rechargeable metal batteries. The advantages and disadvantages with various configurations of photo‐assisted rechargeable metal batteries, and the strategies adopted by researchers to address the pain points will be discussed chronologically in terms of three‐electrode and two‐electrode, respectively (**Figure** **3**). Additionally, the strategies adopted by researchers to accelerate the reaction kinetics of photo‐assisted batteries, enhance the electrochemical performance, and help them move toward practicalization will be summarized. **Table** **2** summarizes the types and properties of representative photo‐assisted rechargeable metal batteries based on diverse photoelectrodes.

**Table 2:** Configurations and performance of representative photo‐assisted rechargeable metal batteries.

Type	Photoelectrode	Light source	Current density [mA cm^−2^]	Cut‐off capacity [mAh cm^−2^]	Cycle	Discharge Voltage [V]	Charge Voltage [V]	RTE 1^st^ /PCE [%]	References
Li‐O_2_	TiO_2_‐N719	1.5 Sun AM	0.016	0.016	4	–	2.72	–	[21]
	g‐C_3_N_4_‐CP	XEF‐501S Xe‐lamp	0.03	0.03	10	1.96	2.74	71	[38]
	Co‐TABQ	300 W Xe‐lamp	0.10	0.50	50	3.12	3.32	94	[39]
	Ag/Bi_2_MoO_6_	300 W Xe‐lamp	50 mA g^−1^	0.25 mAh	500 h	3.05	3.25	93.8	[47]
	NiO	300 W Xe‐lamp	0.01	0.01	60	2.64	2.73	96.7	[48]
	Siloxene	500 W Xe‐lamp	0.075	0.075	100	3.51	1.90	185	[49]
	Siloxene	500 W Xe‐lamp	0.075	–	200	3.72	1.60	230	[52]
	WO_3_	300 W Xe‐lamp	0.02	10 500 mAh g^−1^	200 h	–	–	–	[53]
	TiO_2_	300 W Xe‐lamp	0.02	0.03	30	2.65	2.86	92.6	[42]
	TiO_2_‐Fe_2_O_3_	500 W Xe‐lamp	0.01	0.01	100	3.06	3.24	94.4	[46]
Li‐Ion	TiO_2_	300 W Xe‐lamp	0.02	97.10 mAh g^−1^	10	3.41	2.78	–	[22]
	V_2_O_5_	12 mW cm^−2^	1 A g^−1^	≈100 mAh g^−1^	17	–	–	2.6	[64]
	Cs_3_Bi_2_I_9_	1 Sun	0.05	≈400 mAh g^−1^	3	–	–	0.43	[75]
	CuO	1 Sun	150 mA g^−1^	≈440 mAh g^−1^	150	–	–	0.34	[76]
	Ni/CdS@Ni_3_S_2_	1 Sun	0.5	≈1.05	10	–	–	0.68	[77]
Li–S	CdS‐TiO_2_	0.5 Sun	0.2	1225.0 mAh g^−1^	50	–	–	2.3	[84]
	RGO/CdS	80 mW cm^−2^	0.5 C	1054.0 mAh g^−1^	100	–	–	5.04	[88]
Li‐CO_2_	CsPbBr_3_/Cs_4_PbBr_6_	500 W Xe‐lamp	5 C	679.0 mAh g^−1^	1500	–	–	–	[89]
	In_2_S_3_@CNT/SS	–	0.01	0.01	24	3.14	3.20	98.1	[92]
	Ag‐TiO_2_	400 W UV lamp	0.10	0.10	100	2.49	2.86	86.9	[93]
	TiO_2_	300 W Xe‐lamp	0.01	0.01	30	2.82	2.88	97.9	[96]
	TiO_2_	300 W UV lamp	0.025	–	52	2.25	3.03	74.2	[99]
Li‐I_2_	TiO_2_	1 Sun	0.50	–	–	3.30	2.90	–	[102]
Zn‐Air	α‐Fe_2_O_3_	500 W Xe‐lamp	0.50	0.17	50 h	≈1.15	≈1.64	≈70	[110]
	MnS_2_‐ONT	70 mW cm^−2^	2	799.49 mAh g^−1^	200 h	≈1.2	≈1.60	≈75	[111]
	TiO_2_‐PDTB	90 mW cm^−2^	–	–	33	1.90	0.59	322.03	[118]
	pTTh/CuO_x_	300 W Xe‐lamp	50 mA g^−1^	0.25 mAh	500 h	1.64	0.63	260.32	[119]
	Ni_12_P_5_@NCNT	300 W Xe‐lamp	10	–	320	1.22	1.90	64.2	[7]
	pTTh	500 W LED	0.10	0.24	64 h	1.78	≈2.0	≈89	[8]
Zn‐Ion	V_2_O_5_‐P3HT‐RGO	12 mW cm^−2^	50 mA g^−1^	370.0 mAh g^−1^	–	–	–	≈1.2	[120]
	MnS_2_‐ZnO‐CF	12 mW cm^−2^	100 mA g^−1^	340.0 mAh g^−1^	–	–	–	≈1.8	[121]
	VO_2_‐RGO‐CF	12 mW cm^−2^	200 mA g^−1^	315 mAh g^−1^	–	–	–	≈0.18	[122]
Zn‐I_2_	TiO_2_	320 W UV lamp	0.01	≈40 mAh g^−1^	–	1.2	0.56	–	[127]
Zn‐CO_2_	Cu_2_O/CuCoCr	300 W Xe‐lamp	0.025	0.0125	55 h	1.22	2.07	58.94	[128]
Zn‐Te	CH_3_NH_3_PbI_3_/TiO_2_	250 W Xe‐lamp	100 mA g^−1^	785.0 mAh g^−1^	–	–	–	0.31	[129]
Al‐Ion	MnO_2_	300 W Xe‐lamp	0.1 A g^−1^	531.0 mAh g^−1^	–	–	–	1.2	[130]
Na‐O_2_	TiO_2_	150 W Xe‐lamp	0.015	–	–	≈2.5	≈2.65	≈94.3	[11]

Note: RTE represents the round‐trip efficiency of Li‐O_2_/CO_2_, Zn‐Air/CO_2_, and Na‐O_2_ battery; PCE represents the photo conversion efficiency of Li‐Ion/S, Zn‐Ion/I_2_/Te, and Al‐Ion battery.

### 3.1 Photo‐Assisted Li Metal‐Based Battery

#### 3.1.1 Photo‐Assisted Li‐O_2_ Battery

**Figure 4:**
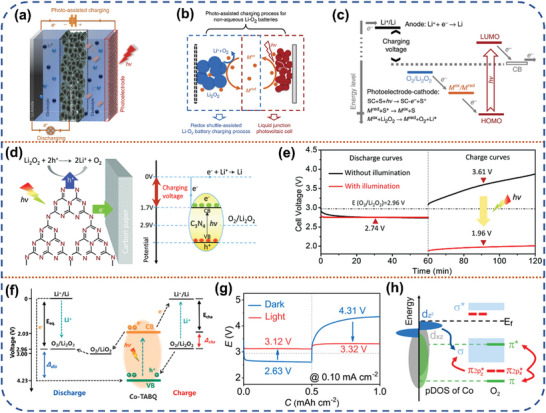
a) The scheme of the three‐electrode solar battery. b) The proposed photoelectrochemical mechanism of the photo‐assisted charging process. c) The energy diagram of the solar battery. Reproduced with permission.^[^ ^21^ ^]^ Copyright 2014, Springer Nature. d) The theoretical potential diagram illustrates the photo‐assisted charge voltage equals the energy difference between the redox potential of the Li^+^/Li couple and CB of g‐C_3_N_4_ (1.7 V). e) The charge/discharge curves of Li‐O_2_ battery with (red line) and without illumination (black line). Reproduced with permission.^[^ ^40^ ^]^ Copyright 2016, Royal Society of Chemistry. f) Reaction mechanism of the photo‐assisted Li‐O_2_ battery with Co‐TABQ. g) Discharge and charge profiles of the Li‐O_2_ battery with and without illumination. h) pDOS of Co in Co‐TABQ and its schematic formation of σ and π bonds with O_2_. Reproduced with permission.^[^ ^41^ ^]^ Copyright 2021, American Chemical Society.

Metal‐air batteries have gained significant attention as a promising alternative due to their considerably higher theoretical energy density compared to current Li‐ion batteries.^[^ ^33^ ^]^ Metal‐air batteries generate electrical energy by undergoing a redox reaction involving the interaction of a metal with atmospheric oxygen. The crucial characteristic of metal‐air batteries lies in the open cell structure of cathode, which facilitates a continuous intake of oxygen from the surrounding atmosphere. This open structure gives metal‐air batteries excellent energy density and many other advantages such as light weight, compactness and low cost.^[^ ^34^ ^]^ Out of numerous anode metals, the Li‐O_2_ battery stands out as a highly promising option owing to its exceptional specific energy density (5200 Wh kg^−1^).^[^ ^35^ ^]^ Unfortunately, the sluggish redox kinetics during the oxidation reaction of Li_2_O_2_ lead to large polarization voltage, which is a key factor hindering the industrialization of Li‐O_2_ batteries.^[^ ^36^, ^37^ ^]^ Despite significant efforts in designing cathode catalysts and developing new redox mediator for Li‐O_2_ batteries, it remains challenging to obtain efficient catalysts that can accelerate the charging and discharging process and reduce the polarization voltage.^[^ ^38^, ^39^ ^]^ In 2014, Wu et al. first proposed a strategy to construct an additional dye‐sensitized photoelectrode on the Li‐O_2_ battery to contribute the high charging voltage needed to charge the Li‐O_2_ battery using solar photovoltage (**Figure** **4**a).^[^ ^21^ ^]^ Specifically, the electrons generated by the dye‐sensitized photoelectrode when exposed to light are transported to the CB of TiO_2_. The holes on the VB of dye molecule can then oxidize the shuttle mediator triiodide ions into iodide ions. These ions are then diffused to the cathode to oxidize the discharge product, Li_2_O_2_. This approach effectively reduces the overpotential for charging the Li‐O_2_ battery (Figure 4b,c). Zhou et al. discovered that, in the absence of redox mediators, C_3_N_4_ photocatalyst could directly oxidize the solid‐state product Li_2_O_2_ by utilizing the photo‐generated holes in the VB of C_3_N_4_ (Figure 4d).^[^ ^40^ ^]^ It resulted in a reduction of the charging voltage of the Li‐O_2_ batteries from 3.61 to 1.96 V (Figure 4e). The two‐electrode system does not require additional photoelectrodes and redox mediator, which can effectively reduce the battery volume and improve the energy density while also avoiding the side reactions and loss mass of active materials due to the presence of redox mediator in the three‐electrode system. Soon, He et al. also presented in their work that TiO_2_ photocatalyst as a photoelectrode can not only directly oxidize Li_2_O_2_ into oxygen and Li ions, making the charging voltage drop from 4.31 to 2.86 V, but also the light radiation will drive the escape of lattice oxygen in TiO_2_, leaving abundant oxygen vacancies to enhance the electron transfer, expediting the discharge procedure.^[^ ^42^ ^]^ Afterward, the two‐electrode system became prevalent in photo‐assisted Li‐O_2_ batteries, and more focus was given to the development of photoelectrode/cathode.^[^ ^43^, ^44^, ^45^ ^]^ As the study of oxidation kinetics at the charging end of photo‐assisted Li‐O_2_ batteries has progressed, attention has also been focused on the discharging process. It is being explored whether photoelectrons generated by photoelectrode can influence the process of oxygen reduction reaction (ORR) in Li‐O_2_ batteries. Xu et al. recently demonstrated that the dense photoelectrons on the surface of Fe_2_O_3_‐TiO_2_ heterojunction under light illumination can effectively regulate the deposition morphology of Li_2_O_2_ by constructing Fe_2_O_3_‐TiO_2_ bifunctional photoelectrodes.^[^ ^46^ ^]^ The photo‐generated holes can, in turn, accelerate the decomposition of Li_2_O_2_ during the subsequent charging process. The photo‐assisted Li‐O_2_ battery exhibits an extremely low polarization voltage of only 0.19 V and good cycling stability, with an ≈86% round‐trip efficiency for 100 cycles. Liu et al. designed a bifunctional catalyst containing cobalt‐tetramine‐benzoquinone (Co‐TABQ), a metal‐organic polymer, to accelerate the reaction kinetics at the cathode (Figure 4f).^[^ ^41^ ^]^ The DFT and experimental results indicate that the Co atom in Co‐TABQ serves as the active site for oxygen reduction. Initially, the oxygen is adsorbed on the surface of Co atom, and subsequently, the π^*^_2p_ orbital of O_2_ receives electrons from the d_z_^2^ and d_xz_ orbitals of the Co atom, leading to its reduction to LiO_2_. The final product, Li_2_O_2_, is then formed (Figure 4g). During the subsequent charging process, the holes in the d_z_^2^ orbital of Co atom promote the breakdown of Li_2_O_2_ into lithium ions and oxygen. Under light irradiation, the discharge voltage can reach 3.12 V and the charging voltage is reduced to 3.32 V (Figure 4h), while maintaining a 94% round‐trip efficiency.

**Figure 5:**
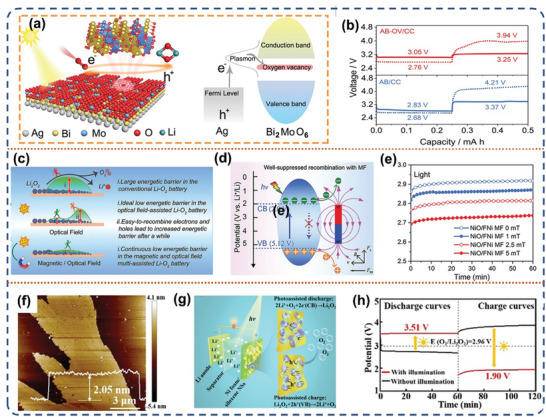
a) Schematic illustration of the AB‐OV/CC photocathode for Li‐O_2_ battery. b) Discharge and charge profiles of the battery at a current density of 50 mA g^−1^, solid lines represent illumination and dash lines represent without illumination. Reproduced with permission.^[^ ^49^ ^]^ Copyright 2021, Wiley‐VCH. c) The diagram shows the decomposition of the discharge products during the charging process (right). d) Schematic diagram of the interaction between the magnetic field and the NiO/FNi under illumination. e) Charge profiles of the battery under an optical field and a magnetic field (MF) with various strengths at 0.01 mA cm^−2^. Reproduced with permission.^[^ ^50^ ^]^ Copyright 2021, Wiley‐VCH. f) Tapping‐mode AFM image of siloxane NSs. g) Schematic diagram of photo‐assisted Li‐O_2_ battery with the siloxane NSs@Ni foam photoelectrode. h) Discharge and charge profiles of the photo‐assisted battery with and without illumination. Reproduced with permission.^[^ ^51^ ^]^ Copyright 2021, Wiley‐VCH.

**Figure 6:**
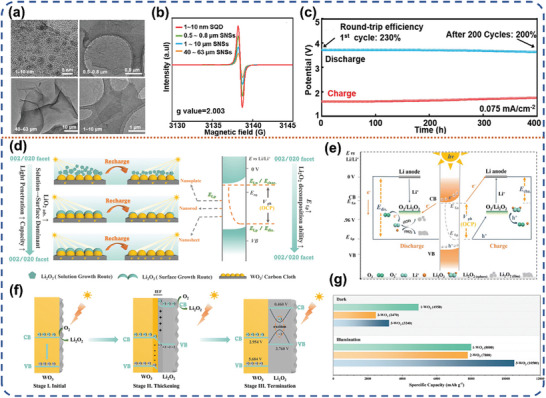
a) TEM images of the different‐sized SNSs and SQD. b) EPR curves of differently sized SNSs AND SQD. c) Long‐time cycle performance of the photo‐assisted Li‐O_2_ battery with SQD photoelectrode under illumination at the current density of 1 mA cm^−2^. Reproduced with permission.^[^ ^52^ ^]^ Copyright 2023, American Chemical Society. d) Schematic of the facet‐controlled Li_2_O_2_ growth routes and WO_3_ photocatalytic in photo‐assisted LOBs. e) The proposed charge/discharge process in the WO_3_/CC cathode under illumination. f) The proposed mechanism for the photo‐assisted discharge process. g) Galvanostatic discharge profiles of the three photocathodes at 50 mA g^−1^ without and with illumination. Reproduced with permission.^[^ ^53^ ^]^ Copyright 2023, Royal Society of Chemistry.

To ensure highly active bifunctional catalysts, it is crucial to improve the migration effectiveness of photo‐generated electron‐hole pairs and inhibit the recombination of photoinduced carriers.^[^ ^47^, ^48^ ^]^ Xu et al. have conducted a series of outstanding works to implement this strategy.^[^ ^49^ ^]^ Constructing an Ag/Bi_2_MoO_6_ hybrid cathode with oxygen defects allows for the injection of hot electrons derived from the localized surface plasma effect into the oxygen‐deficient energy level of Bi_2_MoO_6_ (**Figure** **5**a). This promotes oxygen reduction to generate amorphous Li_2_O_2_ and enhances the discharge plateau voltage to 3.05 V. The highly efficient separation of carriers ensures that photo‐generated holes can rapidly decompose the Li_2_O_2_, resulting in an extremely low charging voltage plateau of 3.25 V (Figure 5b). As a result, the battery exhibits a round‐trip efficiency of 93.8% on the first lap and can cycle for up to 500 h while maintaining a round‐trip efficiency of 70%. Additionally, they proposed a strategy to introduce a magnetic field into a photo‐assisted Li‐O_2_ battery to promote the separation of carriers (Figure 5c).^[^ ^50^ ^]^ The recombination process of excited electron‐hole pairs which generated by NiO/FNi photoelectrode was strongly hindered by the reverse Lorentz force derived from the magnetic field (Figure 5d). The Li‐O_2_ battery is charged at a voltage of only 2.73 V with an energy efficiency of 96.7% attributed to the synergetic effect of light and magnetic fields in the multi‐physics field (Figure 5e). This work suggests the potential application of multi‐physical field, such as magnetic and optical fields, in designing high‐performance energy storage devices. Liu et al. explored the constitutive relationship between the material size and photocatalyst performance. They prepared an oversized siloxane nanosheet through topochemical exfoliation (Figure 5f).^[^ ^51^ ^]^ This ultrathin nanosheet (NS) photocatalyst demonstrated exceptional light trapping ability and low recombination efficiency of carriers. The application of siloxane NS to photo‐assisted Li‐O_2_ batteries resulted in a significant breakthrough in round‐trip efficiency, achieving up to 185%. Furthermore, the cycle life was long‐lasting, maintaining up to 92% efficiency even after 100 cycles. Additionally, a reversible specific capacity of 1170 mAh g^−1^ at 0.75 mA cm^−2^ was achieved (Figure 5g,h). Latest, Liu et al. prepared a series of siloxene materials with varying particle sizes, ranging from a few nanometers to tens of micrometers (**Figure** **6**a).^[^ ^52^ ^]^ Electron paramagnetic resonance (EPR) tests revealed that the siloxene quantum dots (SQD) have the highest abundance of defects (Figure 6b). DFT and experimental results showed that SQD have the highest CB position and the lowest VB value, implying a higher reduction energy level and a stronger oxidation energy level. Meanwhile, the SQD exhibits a higher adsorption energy for oxygen and a lower binding energy with Li_2_O_2_, which enables accelerated oxygen reduction kinetics and rapid Li_2_O_2_ desorption for accelerated Li_2_O_2_ oxidation reaction. Predictably, the SQD based photo‐assisted Li‐O_2_ battery delivered an impressive round‐trip efficiency of 230% at a discharge voltage of 3.72 V and the minimum voltage potential of 1.60 V. The decay rate is only 13% after 200 cycles, with a high round‐trip efficiency of 162% even at a current density of 3 mA cm^−2^ (Figure 6c). Meanwhile, the conformational relationship between lattice structure and catalytic activity was also deeply investigated by Chen et al.^[^ ^53^ ^]^ Through crystal face engineering strategy, they discovered that the Li_2_O_2_ deposition route was shifted from solution growth to the surface growth mode which caused faster redox kinetics when exposing more (002) facets in the WO_3_ photocathode (Figure 6d). The (002) crystal face promotes stronger oxidation of Li_2_O_2_ and brilliant adsorption performance of O_2_^−^/LiO_2_, resulting in an ultra‐low polarization overpotential of 0.07 V. Importantly, the surface growth mode of the WO_3_ photocathode, dominated by the (002) facet, promotes the sustained growth of the Li_2_O_2_ layer up to ≈130 nm thick (Figure 6e,f). An in‐depth investigation shows that a Z‐type heterojunction forms between WO_3_ and the discharge product of Li_2_O_2_ film, which can further stimulate the growth of the Li_2_O_2_ layer. These results demonstrate that (002) facet dominated WO_3_ photoelectrode can achieve a discharge specific capacity of up to 10500 mAh g^−1^ for 200 h of continuous cycling, surpassing the premature death limit caused by surface‐mediated growth (Figure 6g).

The collective efforts of researchers have led to significant advancements in the development of photo‐assisted Li‐O_2_ batteries.^[^ ^6^, ^54^, ^55^ ^]^ High‐efficiency photoelectrode materials, heterojunction bifunctional catalysts, and multi‐physical field coupling have all contributed to the improved performance of the batteries.^[^ ^56^, ^57^, ^58^ ^]^ Specifically, these advancements have led to improvement in the specific capacity, rate performance and cycle ability of the battery.^[^ ^59^, ^60^, ^61^, ^62^ ^]^ Future research should aim to improve the storage and energy efficiency of Li‐O_2_ batteries with the assistance of light fields. It is important to note that testing conditions for these devices are often not uniform, including variations in optical wavelength range and power. Additionally, there is a need to extend the use of these photocathode materials to pouch cell for further advancing the practicalization of photo‐assisted Li‐O_2_ batteries.^[^ ^63^ ^]^

#### 3.1.2 Photo‐Assisted Li‐Ion Battery

**Figure 7:**
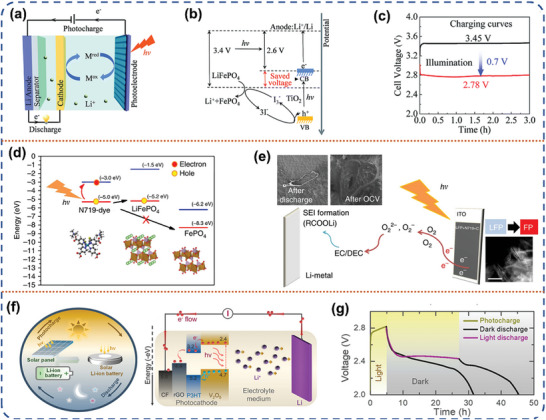
a) Schematic illustration of the photo‐assisted chargeable Li‐ion battery with the three‐electrode system. b) The energy diagram of the photo‐assisted chargeable Li‐ion battery. c)The charge curves of the photo‐assisted batteries with light illumination (red line) and without light illumination. Reproduced with permission.^[^ ^22^ ^]^ Copyright 2015, Royal Society of Chemistry. d) Energy band alignment of the photo‐cathode components. e) The photo‐assisted charging mechanism. Reproduced with permission.^[^ ^66^ ^]^ Copyright 2017, Springer Nature. f) Schematic illustration of the designed photo‐assisted Li‐ion battery concept. g) Photo‐charge for 5 h and galvanostatic discharge in dark and illuminated conditions of the Photo‐assisted battery. Reproduced with permission.^[^ ^67^ ^]^ Copyright 2021, American Chemical Society.

With the advancement of science and technology, the energy density of traditional Li‐ion batteries has nearly reached its maximum theoretical capacity. Lithium metal anode with ultra‐high theoretical specific capacity (3860 mAh g^−1^) has been reemphasized to replace the common graphite‐based material anode.^[^ ^64^, ^65^ ^]^ However, the high charging voltage caused by the kinetic limitation of the Li ion deintercalation behavior from the cathode material structure still persistently plagues the new Li‐ion batteries with lithium metal anode. Photo‐assisted Li‐ion battery system introduces the photovoltage generated by solar energy can help the delithiation behavior of the cathode side, thus reducing the charging voltage. In 2015, Zhou et al. first designed a three‐electrode photo‐assisted Li‐ion battery with an additional TiO_2_ photoelectrode in the LiFePO_4_‐Li battery (**Figure** **7**a).^[^ ^22^ ^]^ With the help of the I^−^/I^3−^ shuttle mediators, the photovoltage can well compensate for the charging voltage during the charging process, reducing the charging voltage to 2.78 V, which is lower than the discharge voltage of 3.41 V (Figure 7b,c). At the same time, the I^−^ redox mediator undergoes oxidation to form I_3_^−^ due to the presence of photogenerated holes, and the I_3_^−^ further oxidizes the LiFePO_4_ to extract Li ions. The reduction in charging voltage saves ≈20% of energy when compared to traditional Li‐ion batteries. On the other hand, a different approach was taken by Song et al., who introduced a Li‐ion battery system with three electrodes that in conjunction with photo‐assistance, in which the redox mediator and the energy storage electrode (SE) LiMn_2_O_4_ material act as mutual electron gaining and electron losing objects for the whole charging and discharging process.^[^ ^68^ ^]^ In particular, they thoroughly investigated the kinetic‐thermodynamic properties of different redox mediators and showed that the copper complex mediator (Cu^+/2+^(dmp)_2_), which is thermodynamically favored but kinetically restricted, exhibits higher energy density and efficiency than the thermodynamically constrained yet kinetically rapid iodine mediator under indoor dark‐light conditions. Its photoelectric conversion efficiency (η_overall_) reaches 11.5% under dim light conditions.

Limited by the complex structure and additional manufacturing cost of the three‐electrode system, the two‐electrode system is increasingly favored by researchers.^[^ ^69^, ^70^, ^71^ ^]^ A two‐electrode photo‐assisted Li‐ion battery system that can directly photo‐oxidize lithium iron phosphate was first proposed by Zaghib et al.^[^ ^66^ ^]^ They used a dye‐sensitized photoelectrode (N719‐Ruthenium‐dye) to generate electron‐hole pairs under photo‐excitation, where the holes assisted the LiFeO_4_ (LFP) cathode in delithiation and the solid electrolyte interface on the anode side was created by utilizing photoelectrons through an oxygen reduction reaction. (Figure 7d). Specifically, the photo‐generated electrons first promote the oxygen reduction, followed by the reaction with the carbonate‐based electrolyte, for which the Li metal surface provides nucleation sites, allowing the lithium carbonate‐based electrolyte derivative component to be deposited as a lithium‐containing compound crystalline SEI, which is redissolved during discharge and does not interfere with the Li‐ion transport process (Figure 7e). This work opens up the possibility of designing photo‐assisted Li‐ion batteries on the basis of a two‐electrode device configuration. Recently, V_2_O_5_ has been used as a versatile cathode material for various metal‐ion batteries due to its open structures, large interlayer spacing, multiple valence states, and reversible structural changes.^[^ ^72^, ^73^, ^74^ ^]^ Volder et al. combined P3HT and rGO additives with V_2_O_5_ nanofibers as photoelectrode for the photo‐assisted Li‐ion batteries.^[^ ^67^ ^]^ During the charging process, the photoelectrons generated by V_2_O_5_ under light excitation jump to the CB. Based on the reasonable energy level structure adjustment, these photoelectrons can be sequentially guided to the surface of the carbon nanofibers through the CB of P3HT and rGO to achieve the successful separation of photo‐generated electron‐hole pairs (Figure 7f), thereafter effectively promote the Li‐ion release process of cathode. Simultaneously, the photoelectrons are conveyed to the lithium anode through the external circuit in order to diminish Li^+^ into metallic Li. This highly efficient separation strategy allows the photo‐assisted Li‐ion battery to be directly charged to 2.87 V under light, with a 57% increase in discharge capacity (Figure 7g). The PCE under 455 nm light and 1 sun irradiation are ≈2.6% and 0.22%, respectively. On the other hand, Liu et al. prepared a two‐electrode photo‐assisted Li‐ion battery by directly using LiV_2_O_5_ as the photocathode material without any additional additives.^[^ ^70^ ^]^ The photo‐assisted fast charging mode enables the battery can be charged to 185 mAh g^−1^ in 5 min at a current density of 2 A g^−1^, which is 270% increase in capacity compared to dark condition. A maximum conversion efficiency of 9% over the complete range is achieved in the individual photo‐charging mode with no voltage applied.

**Figure 8:**
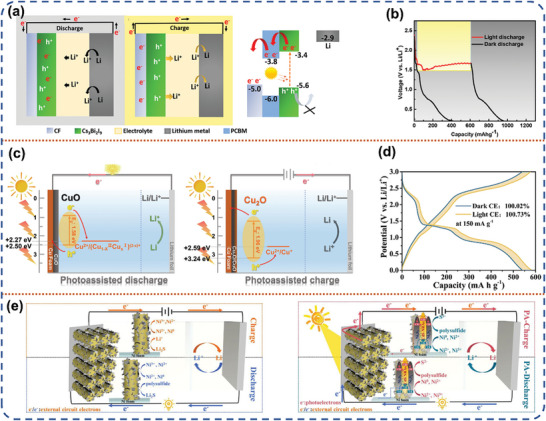
a) Discharge and photo‐charge mechanism respectively of the Cs_3_Bi_2_I_9_ photoelectrode, and the energy level diagram of the perovskite photo‐battery. b) Discharge curves of CF‐PHBATs under light and in the dark. Reproduced with permission.^[^ ^77^ ^]^ Copyright 2021, American Chemical Society. c) The charge and discharge mechanism of photo‐assisted Li‐ion battery based on the Cu/CuO photoelectrode. d) Galvanostatic charge/discharge curves at 4000 mA g^−1^ with dark and light conditions. Reproduced with permission.^[^ ^78^ ^]^ Copyright 2022, Elsevier B.V. e) The working mechanism of Ni/CdS@Ni_3_S_2_ based Li‐ion battery system and photo‐assisted Li‐ion battery system. Reproduced with permission.^[^ ^79^ ^]^ Copyright 2023, Elsevier.

Perovskite materials, because of its unique properties such as tunable bandgap, high carrier mobility, low non‐radiative complexity, broad spectral absorption and long carrier diffusion length is widely applied in the photoelectronic devices and energy storage field.^[^ ^75^, ^76^ ^]^ E. Halpert et al. coupled Cs_3_Bi_2_I_9_ inorganic perovskite halide as a photoelectrode with three different types of collectors: copper, fluorine‐doped tin oxide (FTO) and carbon felt (CF).^[^ ^77^ ^]^ With a transparent collector FTO to elucidate its working mechanism, and composite with CF collector to achieve the most competitive PCE. The photoelectrons generated on the photoelectrode under light irradiation are sequentially delivered to the FTO collector due to the rational energy band arrangement, and the holes on the VB of Cs_3_Bi_2_I_9_ participate in a two‐step discharge process (**Figure** **8**a). Initially, the extensively formed voids exhibit a tendency to expel the intercalated Li ions back into the electrolyte by means of delithiation behavior, which in turn regenerated the pure perovskite cathode. Second, if metallic bismuth is detected at reduced voltage levels, it can photo‐oxidize Bi^0^ to Bi^3+^. After the Li^+^ are repelled from the perovskite structure, the battery voltage is restored and enabling recharging of the device. The photo‐assisted battery using CF as the collector can be directly photo‐charged without external current and then power an external circuit for 2 h, resulting in a PCE of ≈0.43%. The discharge capacity exhibited a significant enhancement, with an increase from 410 to 975 mAh g^−1^ when discharged under light irradiation (Figure 8b).

With such rapid progress in the study of photo‐assisted Li‐ion batteries based on Li‐ion intercalation electrochemistry; little has been reported on photo‐assisted Li‐ion batteries based on the conversion reaction. Recently, Yang et al. reported two works on conversion reaction electrochemistry about photo‐assisted Li‐ion batteries.^[^ ^78^ ^]^ They prepared one‐dimensional copper oxide (CuO) loaded on copper foam collectors with a three‐dimensional structure, which was used as bifunctional photoelectrode to achieve the conversion of sunlight into electricity and then into chemical energy (Figure 8c). The PCE of CuO photoelectrode reached ≈0.34% in the absence of an external power source, and impressively, the charging and discharging capacities of the photo‐assisted battery were enhanced by 64.01% and 60.35% at 4000 mA g^−1^, respectively (Figure 8d). Throughout the charging and discharging procedure, the photo‐generated electrons of CuO drive the interconversion of Cu^2+^ and Cu^+^, which in turn forces the lithium storage to be more complete and increases the specific capacity of the photo‐assisted battery. In addition, they constructed Ni/CdS/Ni_3_S_2_ core‐shell heterojunction nanorod arrays on Ni foam with Ni_3_S_2_ coating on the surface of CdS.^[^ ^79^ ^]^ The Ni/CdS/Ni_3_S_2_ heterojunction was applied as a bifunctional photoelectrode in a photo‐assisted Li‐ion battery, and the overall solar‐to‐electric conversion efficiency of the battery reached 0.11% in the absence of external voltage. Also, the energy conversion efficiency of the photo‐assisted charging and discharging process increased to 3.5% and 2.1%, respectively. The photo‐assisted Li‐ion battery still exhibits a good light response after 250 cycles. In this case, the valence transition of Ni ions promotes an enhanced Li‐ion storage reaction with greater comprehensiveness, which in turn enhances the efficiency of energy storage of Ni/CdS/Ni_3_S_2_‐based Li‐ion battery (Figure 8e). Li‐ion batteries are the epitome of commercial energy storage systems, but the progress of photo‐assisted Li‐ion batteries has been relatively limited. Researchers have primarily focused on enhancing the performance of photo‐assisted Li‐ion batteries using semiconductor photocatalysts, perovskite materials, and heterostructure construction. As can be seen from Table 2, it is clear that the PCE of photo‐assisted Li‐ion batteries are not yet satisfactory. The reasonable design of photoelectrode material matched with suitable electrolyte which is helpful for stabilizing photoelectrode material can improve the extra energy output, thus further improve the PCE of the photo‐assisted energy storage device.

#### 3.1.3 Photo‐Assisted Li–S Battery

**Figure 9:**
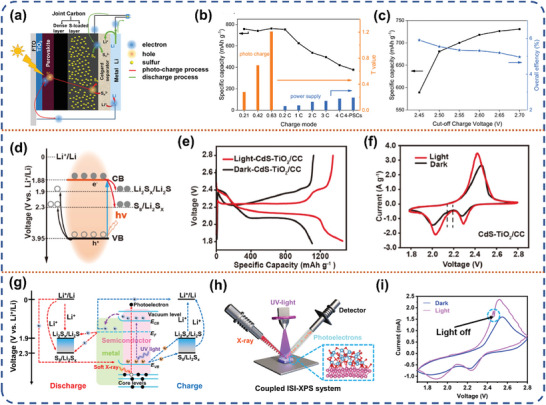
a) Schematic diagram of the fabricated PSC‐Li–S battery. b) Discharge capacities (black line) and T values (blue and yellow columns) of the battery under different charge modes. c) Discharge capacity and overall efficiency of different cut‐off photo‐charge voltage. Reproduced with permission.^[^ ^83^ ^]^ Copyright 2019, Wiley‐VCH. d) Energy diagram of CdS‐TiO_2_/CC and S_8_/Li_2_S versus Li^+^/Li. e) First cycle GDC profiles of the CdS‐TiO_2_/CC batteries with and without the illumination. f) First cycle CV curves of the CdS‐TiO_2_/CC batteries with and without the illumination. Reproduced with permission.^[^ ^84^ ^]^ Copyright 2022, Elsevier Ltd. g) Proposed diagram of charge transfers of photo‐generated carriers in the Li–S battery and working mechanism of ISI‐XPS. h) Schematic illustration of the working configuration of ISI‐XPS. i) CV curves of the NTCNF Li–S battery at a scan rate of 0.2 mV S^−1^ with and without the illumination (λ = 365 nm), the blue circle shows a light switch. Reproduced with permission.^[^ ^85^ ^]^ Copyright 2023, Wiley‐VCH.

Lithium‐sulfur (Li–S) batteries, based on high specific capacity sulfur cathode and lithium anode, have an ultra‐high energy density of 2,600 Wh kg^−1^ and great potential to become the next generation of energy storage system to meet the ever‐increasing global energy production and consumption.^[^ ^28^, ^80^ ^]^ Meanwhile, sulfur is abundant on earth, inexpensive, and environmentally friendly. Despite all these attractive advantages, the complex Li–S electrochemistry, consisting of multi‐step electronic redox reactions and multi‐phase transformations, severely limits the real‐world use of Li–S batteries.^[^ ^81^, ^82^ ^]^ In 2015, Zhou et al. designed a three‐electrode system photo‐assisted Li–S battery based on a Pt/CdS photoelectrode, a lithium metal anode, and a sulfur cathode.^[^ ^23^ ^]^ During the charging process, the CdS photocatalyst generates photoelectron‐hole pairs, the holes oxidize the S^2−^ to polysulfide ions, and the photo‐generated electrons are transported to the surface of Pt metal cocatalyst to reduce protons (H^+^) to hydrogen. This three‐electrode photo‐assisted Li–S battery system simultaneously achieves the goals of solar‐chemistry energy‐electric energy conversion and photocatalytic splitting of water. The device achieved a discharge capacity of 199 mAh g^−1^ following a 10 min photo‐charging period, surpassing the majority of conventional cathode materials used in Li‐ion batteries. Notably, this photo‐assisted device interned where a specific capacity of 280 mAh g^−1^ after 2 h of solar irradiation. At the same time, the corresponding hydrogen production rate was 1.02 mmol g^−1^ h^−1^. On the other hand, Gao et al used the strategy of integrating perovskite solar cell to charge Li–S batteries (**Figure** **9**a).^[^ ^83^ ^]^ To be more specific, a high‐energy Li–S battery is continuously charged by three perovskite solar cells that are assembled in sequence on a single substrate, reaching the achievement of converting solar energy directly into chemical energy. Compared with the power supply mode, the discharge capacity of the photo‐assisted Li–S battery in the photo‐charging mode is positively correlated with the current density, indicating that the adjustable charging mode based on solar charging has a good charging efficiency similar to the dual‐mode charging process of Li‐ion batteries (Figure 9b). This photo‐assisted Li–S battery demonstrates an energy conversion efficiency of up to 5.14%, while outputting a specific capacity of up to 750 mAh g^−1^ at 2 C in the fast photo‐charging mode (Figure 9c). In comparison, the Li–S battery has a specific capacity of only 535 mAh g^−1^ at power mode.

Recently, a pioneering work on photo‐assisted Li–S batteries was reported by Guo et al.^[^ ^84^ ^]^ They designed and fabricated a CdS‐TiO_2_/carbon cloth multifunctional photocathode with lithium metal to form a two‐electrode system photo‐assisted Li–S battery, achieving 100% energy efficiency. Unlike previous photo‐assisted Li–S batteries, which are limited to optimizing the battery charging process, this multifunctional photocathode can simultaneously accelerate the sulfur reduction reaction (SRR) and the sulfur evolution reaction (SER) (Figure 9d). This CdS‐TiO_2_ heterostructure can effectively absorb light and enhance the separation efficiency of photo‐generated electron‐hole pairs, which in turn improves the electrochemical performance of Li–S batteries through photocatalysis, photo‐conductive and photo‐charging effects. The photo‐generated electrons accelerate the sulfur reduction kinetics and lower the reaction energy barrier of lithium polysulfides (LiPSs) to Li_2_S, while the photo‐generated holes are responsible for oxidizing Li_2_S during charging process and facilitating the conversion of Li_2_S to LiPSs. As a result, the deposition potential of Li_2_S is increased by 70 mV compared to that in the absence of light, while the voltage for charging experiences a decrease of ≈0.1 V. (Figure 9e,f). The photo‐conductive effect involves the enrichment of abundant photo‐generated carriers formed on the cathode surface to further pearlized the electrochemical reaction kinetics. The photo‐charging effect directly recharges the Li–S battery to 608 mAh g^−1^, achieving an energy conversion efficiency of 2.3%. This study introduces a novel avenue for utilizing photo‐assisted technology in Li–S batteries, which can also be extended to other energy storage systems like sodium‐sulfur and magnesium‐sulfur batteries.^[^ ^86^, ^87^ ^]^ In addition to further improving the solar energy conversion efficiency to facilitate the development of photo‐assisted Li–S batteries, the fundamentals of photo‐assisted batteries can be used to elucidate the catalytic mechanism of Li–S batteries. In the latest work published by our group, we have identified the sulfur reduction and Li_2_S oxidation sites in the Li–S electrochemistry at the molecular level by coupling the photo‐assisted effect with in situ X‐ray photoelectron spectroscopy (ISI‐XPS) (Figure 9g,h).^[^ ^85^ ^]^ The enhanced selective electrocatalytic effect in the designed photo‐assisted Li–S battery was directly observed at the atomic level, and the catalytic center of the Li–S system was identified by in situ observation of the directional migration of electron‐hole pairs in the energy band structure of the electrocatalysts, which elucidated the selective electrocatalytic mechanism of Li–S electrochemistry (Figure 9i). Remarkably, the pouch cell featuring a cathode composed of S/NTCNF demonstrates remarkable flexibility and remains functional even in challenging operational environments. Our study presents a viable approach for fabricating electrocatalysts with selective pair sites capable of facilitating the reduction of LiPSs and the decomposition of Li_2_S. This approach offers a universal method to enhance the comprehension of bidirectional sulfur electrochemistry at a deeper level.

In addition, Yang et al. used RGO/CdS as the photo‐rechargeable integrated lithium‐sulfur cathode, proved the effectiveness of the photocatalytic effect in catalyzing the conversion of LiPSs through the adsorption experiments.^[^ ^88^ ^]^ The experimental data show that the photocatalytic effect can significantly reduce the polarization voltage of Li–S batteries. The battery performance of the photo‐rechargeable integrated lithium‐sulfur batteries (PRLSBs) increased by 113.3% at a high rate of 1 C. After 1.5 h of photo‐charging, the PRLSBs can continuously supply energy for 21 h. At the same time, the photo‐assisted Li–S battery with perovskite quantum dots loaded on MOF materials which developed by Chen et al. can stably cycle for 1500 cycles at a high rate of 5 C, and the capacity fading rate is only 0.022%, per cycle.^[^ ^89^ ^]^ It fully demonstrates the bright development potential of photo‐assisted Li–S batteries. The key advantage of photoelectrically responsive materials is that high energy carriers can directly participate in the redox reaction of sulfur species, thereby intuitively accelerating Li–S electrochemistry. In view of the most complex 16 electrons reaction of sulfur, the stability of photoelectrode materials requires special attention. Therefore, in practical applications, stability factors must be carefully considered and reasonable measures must be taken to achieve optimal battery performance and realize the utilization of solar energy.

#### 3.1.4 Photo‐Assisted Li‐CO_2_ Battery

**Figure 10:**
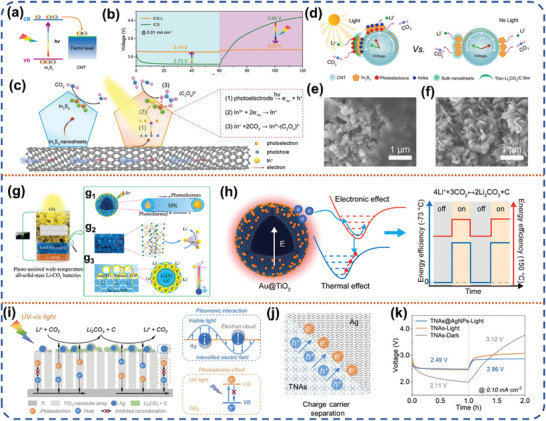
a) Bind diagram of the In_2_S_3_@CNT/SS (ICS). b) Discharge and charge curves of the ICS‐based Li‐CO_2_ battery with and without illumination at 0.01 mA cm^−2^. c) Illustrations of the working mechanism for the light‐induced discharging process. d) A model illustrating the effect of solar energy on the final morphology and decomposition of the deposited discharge products. e,f) SEM images of the first discharged and recharged ICS cathode with and without illumination. Reproduced with permission.^[^ ^93^ ^]^ Copyright 2020, Wiley‐VCH. g) Schematic representation of the architecture for all solid‐state Li‐CO_2_ battery with an integrated structure of the solid‐state Au@TiO_2_ spheres deposited on the porous LAGP cathode. g_1_) Scheme for the photoelectronic and photothermal effect on the Au@TiO_2_ spheres. g_2_) Li^+^ transfer in the crystal structure in LAGP. g_3_) Working principle of the integrated structure under the wide temperature scale is ≈−73–150 °C. h) Mechanism for the photoelectronic effect and photothermal effect of Au@TiO_2_ on the electrochemical reaction. Reproduced with permission.^[^ ^94^ ^]^ Copyright 2022, American Chemical Society. i) Working mechanism of the dual‐field assisted Li‐CO_2_ battery via synergistic photoelectric effect and plasmonic interaction. j) Complementary schematics of the charge carrier migration inside the TNAs@AgNPs. k) Discharge/charge voltage profiles at the first cycle. Reproduced with permission.^[^ ^95^ ^]^ Copyright 2022, Wiley‐VCH.

Since the industrial revolution, human beings have been emitted increasing amounts of heat‐absorbing greenhouse gases, such as carbon dioxide, into the atmosphere. It has aggravated the greenhouse effect, leading to a series of problems that have raised concerns worldwide. Rechargeable lithium‐carbon dioxide (Li‐CO_2_) batteries have been made to mitigate CO_2_ emissions and decrease reliance on fossil fuels by converting CO_2_ into sustainable electricity.^[^ ^90^, ^91^ ^]^ Despite their high energy density of 1,876 Wh kg^−1^, the non‐conducting nature of the discharge product, Li_2_CO_3_, results in sluggish kinetics, leading to high polarization voltages and low energy densities.^[^ ^92^ ^]^ To address this issue, further research is needed to improve the CO_2_ reduction reaction (CDRR) and the CO_2_ evolution reaction (CDER). Recently, Xu et al. designed a In_2_S_3_@CNT/SS (ICS) bifunctional photocathode and applied it in a photo‐assisted Li‐CO_2_ battery.^[^ ^93^ ^]^ The photo‐assisted Li‐CO_2_ battery exhibited a discharge voltage of 3.14 V, surpassing the thermodynamic threshold of 2.8 V, while maintaining an exceptionally low charge voltage of 3.20 V. The remarkable performance showcases an impressive round‐trip efficiency reaching up to 98.1%. (**Figure** **10**a,b). Photo‐generated electrons play a key role in the light‐introduced discharge process. Specifically, the ICS is excited to produce separated electron‐hole pairs, and then the photo‐generated electrons will reduce In^3+^ to In^+^, due to the fact that the reduction potential of In^3+^/In^+^ is more positive than the CB of ICS (Figure 10c). Simultaneously, the holes will be conducted through the conducting carbon nanotube (CNT) network to the external circuit to compound with the electrons. Subsequently, CO_2_ was adsorbed on the surface of ICS and reduced to In^3+^‐C_2_O_4_^−^ by In^+^. This strategy increases the number of nucleation sites for discharge product (Li_2_CO_3_) due to sufficient photoelectron aggregation on the surface of ICS. Consequently, the acceleration of Li^+^ transportation leads to modulation of the deposition process for Li_2_CO_3_, resulting in the formation of a thin film Li_2_CO_3_/C product on the surface of the electrode. (Figure 10d,e). In contrast, the Li‐CO_2_ battery without light‐involution formed a nanosheet morphology Li_2_CO_3_ on the cathode surface due to sluggish kinetics (Figure 10f), resulting in slow growth of Li_2_CO_3_.

Meanwhile, in another work, they demonstrated that photoelectron aggregation on TiO_2_/carbon cloth (CC) photoelectrode can modulate the deposition of Li_2_CO_3_ discharge product, relevant to the stimulation of the followed CO_2_ evolution reaction.^[^ ^96^ ^]^ This photo‐assisted Li‐CO_2_ battery achieved an ultra‐low charging voltage of 2.8 V and a cycle time over 60 h, with a remarkable energy efficiency rate of 97.9% in the presence of light. Metal‐organic frameworks (MOFs) are specifically designed to meet the desired needs of photo‐assisted Li‐CO_2_ batteries by rational selection of building blocks due to their tunability. Lan et al. assembled single Co‐N_4_ sites, Mn ions and phthalocyanine ligands into photosensitive MOF‐based nanosheets with the following points: 1) the phthalocyanine molecule possesses a broad and robust capacity for absorbing light due to its conjugated structure; 2) the Co‐N_4_ sites in phthalocyanine have been shown to have miles for titanium dioxide reduction reactions, including CO_2_ activation and Li_2_CO_3_ growth; 3) the Mn sites in the MOFs have a positive effect on the CO_2_ evolution reaction; and 4) the morphology of the nanosheet enables a higher exposure of active sites, leading to an enhancement in catalytic efficiency.^[^ ^97^ ^]^ Based on this, the phthalocyanine‐based metal‐organic framework nanosheets (CoPc‐Mn‐O) photocathode enables a high round‐trip efficiency of 98.5%, an ultra‐low polarization voltage of 0.05 V and a good cycling stability over 60 h. Highly active photo‐assisted cathode materials can cause decomposition and depletion of the organic liquid electrolyte, which limits the cycle life of photo‐assisted batteries.^[^ ^98^ ^]^ Therefore, a more stable and safe solid‐state electrolyte system is a viable alternative. For instance, Xu et al. proposed an all‐solid‐state Li‐CO_2_ battery comprising a lithium cathode, an ultrathin and dense Li_1.5_Al_0.5_Ge_1.5_(PO4)_3_ (LAGP) all‐solid‐state electrolyte layer (100 µm thick) and an Au@TiO_2_ photocathode loaded on the LAGP (Figure 10g).^[^ ^94^ ^]^ This structural design enables safe operation and stability over a wide temperature range. The Au@TiO_2_ heterojunction photocathode utilizes the surface plasmon resonance effect (SPR) to capture and stratify broad‐spectrum solar energy, enabling sustained self‐heating and photovoltaic activity (Figure 10g1). Additionally, the LAGP ceramic structure exhibits exceptional Li‐ion conductivity, thermal conductivity, and thermal stability (Figure 10g2). Finally, the LAGP‐based skeleton exposes more three‐phase boundaries to improve the efficiency of light absorption and dispersion (Figure 10g3,h). The all‐solid‐state Li‐CO_2_ battery achieves an ultra‐low polarization of 0.25 V and a round‐trip efficiency of 92.4%. At a temperature as low as −73 °C, the self‐heating effect by transforming sunlight into thermal energy still delivers a low polarization voltage of 0.6 V. Peng et al. used plasma effect‐assisted TiO_2_ photoelectrode to improve the separation efficiency of photo‐generated carriers and inhibit the recombination of electron‐hole pairs.^[^ ^95^ ^]^ Under light radiation, the TiO_2_ nanotube arrays (TNAs) generated a large number of energetic photoelectrons and holes. The incident light caused the collective oscillations of Ag nanoparticles, resulting in effective scattering and an increased electric field. It enhances the separation and transfer of photo‐excited carriers for CO_2_ redox reactions (Figure 10i,j). By synergistically utilizing the photoelectric effect of TNAs alongside the plasmonic interaction of silver nanoparticles, the innovative dual‐field‐assisted strategy exhibits significant potential in augmenting the electrochemical capabilities of Li‐CO_2_ batteries. The prepared dual‐field‐assisted battery achieves an ultra‐low charging voltage of 2.86 V and maintains an efficiency of 86.9% after 100 cycles (Figure 10k). This strategy offers a versatile and efficient approach to achieving optimal performance of Li‐CO_2_ batteries. Furthermore, its application can extend to other categories of metal‐air batteries.

Latest, Meng et al. prepared a mixed‐phase TiO_2_ consisting of rutile and anatase phases by optimizing the annealing temperature.^[^ ^99^ ^]^ This mixed‐phase heterogeneous structure constitutes a typical type II heterojunction photoelectrode and was applied into a photo‐assisted Li‐CO_2_ battery system. Under ultraviolet irradiation, rutile, and anatase TiO_2_ generate electron‐hole pairs simultaneously. The type II heterojunction energy band arrangement allows electrons in the CB of rutile to inject into the CB of anatase, accelerating the separation of photo‐generated carriers. The mixed‐phase TiO_2_ photocathode delivered an area‐specific capacity of 3001 uAh cm^−2^ under UV irradiation, which is higher than the 1970 uAh cm^−2^ achieved in the absence of light. Additionally, the initial charging voltage of this Li‐CO_2_ battery was reduced from 4.53 V to 3.03 V. At present, the research focus of photo‐assisted Li‐CO_2_ batteries are still on improving the round‐trip efficiency, and the problem of cycle stability still persists. Improving the adsorption capacity of the photoelectrode to CO_2_ is the first step to accelerate the CO_2_ redox reaction. Reasonable photocatalyst design includes plasma effect and built‐in electric field, which can effectively reduce the energy barrier of CO_2_ redox reaction. Constructing the photoelectrode structure with synergistic adsorption and efficient photocatalytic capabilities is an effective strategy to promote the development of photo‐assisted Li‐CO_2_ batteries.

#### 3.1.5 Photo‐Assisted Li‐I_2_ Battery

**Figure 11:**
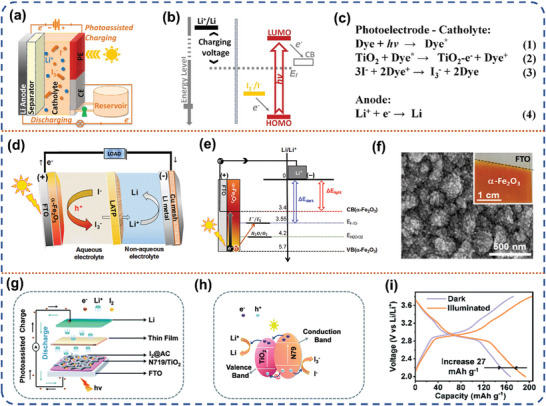
a) Schematic of a Li‐I_2_ solar flow battery (SFB) device with the three‐electrode configuration. b) Energy diagram for the photo‐assisted charging process. c) Photoelectrochemical reaction of the half‐reactions. Reproduced with permission.^[^ ^102^ ^]^ Copyright 2015, American Chemical Society. d) Illustrations of photo‐assisted charge process in aqueous Li‐I_2_ cells using a hematite photoelectrode (α‐Fe_2_O_3_/FTO substrate). e) The corresponding energy diagram describing different open circuit voltage (OCV) under light and dark. f) Top‐view SEM and digital (inset) images of hematite. Reproduced with permission.^[^ ^103^ ^]^ Copyright 2016, American Chemical Society. g) Schematic of the photo‐assisted rechargeable Li‐I_2_ battery. h) The working mechanism of the photo‐assisted charging process. i) GCD curves at 100 mA g^−1^ under dark and illuminated conditions. Reproduced with permission.^[^ ^104^ ^]^ Copyright 2022, Royal Society of Chemistry.

Rechargeable lithium‐iodine (Li‐I_2_) batteries are a promising alternative due to their high discharge plateau voltage (3.65 V vs Li/Li^+^), considerable specific capacity (211 mAh g iodine^−1^), and the abundant iodine resources in seawater (55 ug L^−1^).^[^ ^100^, ^101^ ^]^ The iodine reactive species (I^−^, I_3_^−^, and I_5_^−^) are dissolved in liquid organic electrolytes in direct contact with the electrodes. It is favorable for the catalysts in the photoelectrodes to directly catalyze the redox reactions and reduce the interfacial mass transfer resistance, thereafter enhancing the conversion efficiency of solar energy. Wu et al. reported a liquid photo‐assisted Li‐I_2_ battery.^[^ ^102^ ^]^ The integration of a Li‐I_2_ redox flow battery and a dye‐sensitized solar cell is achieved through the utilization of the I_3_^−^/ I^−^ redox pair in this device. It enables the concurrent transformation and retention of solar energy. The Li‐I_2_ battery has a three‐electrode configuration consisting of a lithium metal anode, a Pt counter electrode and a dye‐sensitized TiO_2_ photoelectrode (**Figure** **11**a). The counter electrode and photoelectrode are in direct contact with a flowing I_3_^−^/I^−^ redox electrolyte, which is pumped inside the device through a reservoir connected to the counter electrode. The discharge process for this battery bears resemblance to the conventional Li‐I_2_ batteries. During the discharging process, Li metal is electrochemically oxidized to lithium ions while I_3_^−^ is reduced to I^−^ at the counter electrode, releasing electrical energy. During the charging process, dye molecules are irradiated with light, which injects photoelectrons into the CB of TiO_2_. This process caused I^−^ to be oxidized to I_3_^−^ by photo‐generated holes, and consequently, the dye molecules are regenerated (Figure 11b,c). At 1 sun AM with 1.5 irradiation, the photo‐ assisted Li‐I_2_ battery was recharged at 2.90 V, which is less than the discharge voltage of 3.30 V. Byon et al. used hematite (α‐Fe_2_O_3_) as a photoelectrode and lithium metal as an anode to create a low‐cost, highly efficient two‐electrode photo‐assisted Li‐I_2_ battery (Figure 11d).^[^ ^103^ ^]^ The hematite material has high visible light absorption efficiency and good stability. Under light irradiation, hematite generates electrons‐hole carriers. The photo‐generated electrons jump to the CB of hematite, and the quasi‐Fermi energy levels of the remaining carriers on the VB align with the I^−^/I_3_^−^redox voltage (Figure 11e,f). The stabilized hematite undergoes repeated photovoltaic reactions in a deep discharge cycle without significant degradation or photo‐corrosion. At a current density of 0.075 mA cm^−2^, the energy efficiency of the photo‐assisted charging process was 95.4%, which is ≈20% higher than the efficiency without light. Recently, Bao et al. developed a photocathode (I_2_@AC/N719‐dye/TiO_2_) for photo‐assisted Li‐I_2_ batteries using iodine monomers compounded with activated carbon, N719 dye‐TiO_2_ (Figure 11g).^[^ ^104^ ^]^ The bandgap gradient between N719 and iodine facilitates the transfer of photo‐generated electrons, resulting in the storage of solar energy in the Li‐ion storage system (Figure 11h). Furthermore, the light‐trapping materials, primarily iodine monomers and N719 dye is not limited to enhancing electron transfer in the redox mechanism within Li‐I_2_ batteries; they also hinder the diffusion of iodine by promoting the rapid formation of lithium iodide. Compared to the absence of light irradiation condition, the integrated photo‐assisted Li‐I_2_ battery increases the charge/discharge capacity from 171/166 to 204/193 mAh g^−1^ (Figure 11i).

### 3.2 Photo‐Assisted Zn Metal‐Based Battery

#### 3.2.1 Photo‐Assisted Zn‐Air Battery

**Figure 12:**
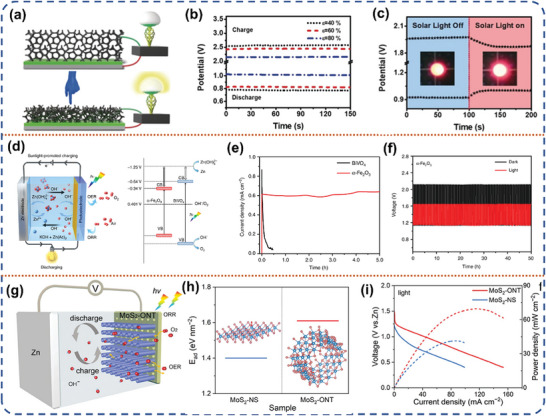
a) Schematic structure and working principle of smart rechargeable Zn‐air battery (SRZAB) under compression. b) Discharge/charge potential curves under different compressive strains. c) Galvanostatic charge/discharge curves of SRZAB in the dark and under sunlight (300 mW cm^−2^, 1 mA cm^−2^). Reproduced with permission.^[^ ^24^ ^]^ Copyright 2019, Wiley‐VCH. d) The scheme of the basic structure and working principle of the photo‐assisted rechargeable Zn‐air battery and the proposed mechanism of the photo‐assisted charging process under solar light illumination. e) Current density‐time curves of the BiVO_4_ and α‐Fe_2_O_3_ photoelectrodes under illumination at 1.23 V versus RHE. f) Cycling performance of photo‐assisted rechargeable Zn‐air battery in the dark and under illumination with α‐Fe_2_O_3_ air photoelectrodes, at a current density of 0.5 mA cm^−2^. Reproduced with permission.^[^ ^110^ ^]^ Copyright 2019, Springer Nature. g) Scheme of the photo‐assisted Zn‐air battery with MoS_2_‐ONT cathode. h) Diagram of oxygen adsorption energy, the inset is the corresponding adsorption structure. i) Discharge polarization and power density curves of the photo‐assisted Zn‐air batteries with MoS_2_‐ONT and MoS_2_‐NS cathodes with light on, respectively. Reproduced with permission.^[^ ^111^ ^]^ Copyright 2023, Wiley‐VCH.

Since the natural safety comes from the use of aqueous, non‐flammable electrolytes and the global abundance of zinc (≈300 times more than lithium), zinc‐air (Zn‐air) batteries have great potential as an alternative to Li‐air batteries. While the theoretical specific energy density of 1,084 Wh kg^−1^ falls below that of Li‐air batteries, it remains four times greater than the current Li‐ion batteries.^[^ ^20^, ^105^ ^]^ Furthermore, the rapid growth of Zn‐air batteries to cater to a vast market's energy demands can be attributed to their numerous advantages such as cost‐effectiveness and affordability, minimal equilibrium potential, consistent discharge voltage, extended lifespan and eco‐friendliness.^[^ ^106^ ^]^ However, there are still some major challenges that need to be addressed for Zn‐air batteries. One of the main difficulties is the high charging overpotential, which is due to the fact that the actual charging voltage is often higher than 2 V, although the calculated theoretical voltage is 1.65 V.^[^ ^107^, ^108^, ^109^ ^]^ In 2017, Dai et al. designed a photosensitive bifunctional electrocatalyst for application in photo‐assisted Zn‐air batteries.^[^ ^7^ ^]^ This bifunctional electrocatalyst consists of Ni_12_P_5_ nanoparticles coupled with N‐doped carbon nanotubes (NCNT) to form a p‐n junction, in which the oxygen reduction reaction (ORR) and the oxygen evolution reaction (OER) are catalyzed by the n‐type NCNT and p‐type Ni_12_P5 active sites, respectively. Under light irradiation, Ni_12_P_5_ is photo‐excited to generate electron‐hole pairs, and the transfer of electrons occurs on the surface of NCNT to accelerate oxygen reduction via the built‐in electric field of the p‐n junction, while the holes remain in Ni_12_P_5_ to promote water oxidation. The photo‐assisted Zn‐air battery features a low polarization voltage of ≈0.75 V and an impressive cycling stability of more than 500 cycles, accompanied by a remarkably reduced charging voltage of 1.90 V and an elevated discharging voltage of 1.22 V. As a result, the voltage polarization is decreased from 0.75 to 0.68 V, resulting in an improvement in the round‐trip efficiency from 61.3% to 64.2% when exposed to light conditions. Besides, Li et al. prepared a polymer semiconductor polytrithiophene (pTTh) photocathode material to convert solar energy into electricity in a direct manner.^[^ ^8^ ^]^ Under the light illumination, photoelectrons generated in the CB of pTTh are injected into the π_2p_^*^ orbitals of oxygen to reduce itself to HO_2_^−^ and finally decouple to OH^−^, thus react with Zn at the anode side to form ZnO. The discharge voltage is greatly increased to 1.78 V and cycled for ≈64 h without degradation. Distinguishing from a single light‐field‐introduced Zn‐air battery, Yu et al. present the first high‐performance integrated haptic and sunlight multi‐stimulus‐responsive all‐solid‐state smart rechargeable Zn‐air battery (SRZAB) (**Figure** **12**a).^[^ ^24^ ^]^ This unique SRZAB was realized using an integrated metal‐free, multi‐sensing air electrode (MSAE) and carbon nanotubes into a catalytic ink, which was subsequently cis‐selectively integrated into a macroscopic polyurethane foam. MSAE integrates bifunctional ORR/OER catalytic activity, pressure sensitivity, photothermal and photovoltaic conversion effects into a single electrode. The SRZAB was able to provide continuous voltage output when a constant voltage load was applied, and the polarization voltage was reduced from 1.8 to 1.1 V (Figure 12b). Furthermore, with the additional application of light field, the charge voltage was reduced from 1.96 to 1.88 V and the discharge voltage was increased from 0.92 to 1.0 V, resulting in an increase in energy efficiency from 46.9% (in dark mode) to 53.2% (Figure 12c). Consequently, this “all‐in‐one” MSAE can act as a fluidic reversible oxygen electrode and stimulation device simultaneously, thereby conferring excellent battery performance, self‐conditioned charging and discharging and versatile utility upon the SRZAB. These include intelligent power management modes for compressible power supplies, self‐powered pressure and optical sensors, and photo‐electrochemical energy systems. What is the constitutive relationship between the energy band structure and the catalytic performance of the photocathode? Hu et al. provide an answer in their work.^[^ ^110^ ^]^ They used two semiconductor catalysts, α‐Fe_2_O_3_ and BiVO_4_, as a demonstration. BiVO_4_ with a lower VB position has a stronger oxidizing ability to oxidize O_2_ to OH^−^ and also exhibits a higher initial photocurrent density, but its catalytic stability is poor due to severe photo‐corrosion factors. Although it has the potential to decrease the charging voltage of the photo‐assisted Zn‐air battery to 1.20 V, it is unable to catalyze the OER reaction in a sustained manner (Figure 12d,e). On the contrary, α‐Fe_2_O_3_, with moderate oxidizing ability and good stability, not only effectively reduces the charging potential to 1.40 V, but also continuously catalyzes the OER reaction, supporting almost no decay for 50 h (Figure 12f). It is also interesting to note that the actual potential difference (≈0.23 V) of the photo‐assisted devices based on two different photoelectrodes is well stabilized with the predicted theoretical value (0.20 V). This work demonstrates that having more negative CB and moderate VB positions can effectively improve the energy efficiency of photo‐assisted Zn‐air batteries. Latest, Xu et al. constructed a kind of 1D ordered MoS_2_ nanotube materials (MoS_2_‐ONT) by the chemical vapor deposition (CVD) method(Figure 12g).^[^ ^111^ ^]^ The 1D MoS_2_‐ONT material with the property of restricted mass transfer can prolong the duration of photo‐induced carriers and overcome the challenge of rapid electron‐hole recombination. The confined space in the form of a tube not only facilitates the separation of carriers, but also boosts the aggregation of charges and expedites the activation process of oxygen molecules. Time‐resolved photoluminescence spectroscopy and Kelvin probe force microscopy reveal that this vertically ordered 1D photo‐responsive nanoreactor gently consolidates the accelerated separation of photo‐generated carriers and efficiently salts their lifetime. In addition, the accelerated ORR kinetics were demonstrated by the density functional theory (DFT) analysis (Figure 12h). A record ORR reaction kinetics of 70 mW cm^−2^ was realized in the photo‐assisted Zn‐air battery under light condition (Figure 12i).

**Figure 13:**
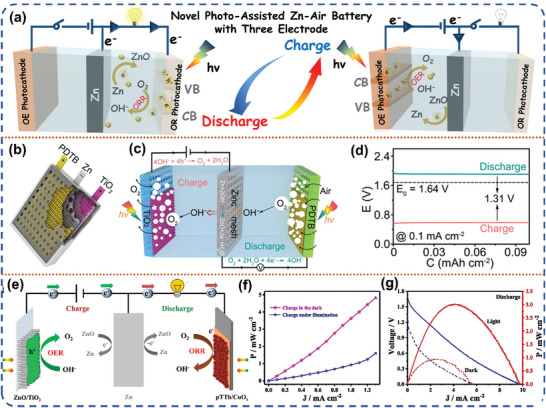
a) Working principle of the discharging and charging process in the novel photo‐assisted Zn‐air battery with three‐electrode. Here, the OR photocathode and OE photocathode, sandwiching the Zn anode in the middle to form a sandwich structure that accelerates the oxygen evolution reaction (OER) and the oxygen reduction reaction (ORR), respectively. b) Prototype of the pouch cell. c) Cross section of photo‐assisted Zn‐air battery. d) Discharge/charge profiles at 0.1 mA cm^−2^ under illumination (365 nm, 90 mW  cm^−2^). Reproduced with permission.^[^ ^118^ ^]^ Copyright 2020, Wiley‐VCH. e) The energy conversion process of the photo‐assisted charge and discharge of the photo‐rechargeable Zn‐air battery (PRZAB). f) The actual required power for charging ZnO/TiO_2_‐Zn‐Ptth/CuO_x_ battery in the dark and under illumination. g) The discharge polarization curves of the PRZAB tested in the dark and under illumination. Reproduced with permission.^[^ ^119^ ^]^ Copyright 2023, Elsevier.

With the deepening of research on photo‐assisted batteries, the traditional three‐electrode system has gradually faded into the background due to the disadvantages including one‐way charging mode, complex structures and high cost.^[^ ^112^, ^113^, ^114^ ^]^ Recently, a novel, unique and customized photo‐assisted three‐electrode system has been developed exclusively for Zn‐air batteries.^[^ ^115^, ^116^, ^117^ ^]^ As shown in **Figure** **13**a, two photocathodes (OR photocathode and OE photocathode) are placed on both sides of the Zn metal anode to form a typical sandwich structure. During the charging process, OE photocathode targets the oxygen evolution reaction, accelerating the oxidation of OH^−^ and releasing oxygen. During the subsequent discharging process, OR photocathode is responsible for catalyzing the oxygen reduction reaction to produce OH^−^. This separate dual photocathode allows for independent and orderly division of labor of the catalysts without interfering with each other, focusing on a single catalytic reaction kinetics. Recently, Li et al. constructed two photocathode materials, poly(1,4‐di(2‐thienyl)) benzene (PDTB) and TiO_2_, aiming the oxygen reduction reaction and oxygen evolution reaction, respectively (Figure 13b,c).^[^ ^118^ ^]^ They are grown in situ on the surface of carbon paper to promote the cathode reaction. During the photo‐discharging process, the PDTB catalyst was used to excite the ORR coupled to the Zn cathode. Throughout the following photo‐charging procedure, the photo‐generated holes derived from TiO_2_ will oxidize OH^−^, which then release oxygen into air. This specific photoelectrode design enables the Zn‐air battery to achieve an exceptionally high discharge voltage of 1.90 V and an unparalleled low charge voltage of 0.59 V (Figure 13d). Latest, Lin et al. reported a three‐electrode configuration photo‐assisted Zn‐air battery composed of ZnO/TiO_2_, Zn‐metal and polyterthiophene (pTTh)/CuO_x_.^[^ ^119^ ^]^ Since the illumination, the type II ZnO/TiO_2_ heterojunction surface undergoes the separation of photogenerated electron‐hole pairs, and the high energy holes oxidized hydroxide to produce H_2_O and O_2_ (Figure 13e). During this period, the photovoltage supplements the potential required for charging to 0.63 V, which is a drastic reduction compared to the current charging voltage of the Pt/C catalyst (1.79 V). At the same time, the subsequent discharging process results in the release of chemical energy which was converted from solar energy. As for the photo‐assisted discharging process, the hot electrons generated by pTTh/CuO_x_ can greatly enhance the kinetics of oxygen reduction reaction and achieve a notable boost in discharge voltage up to 1.64 V, surpassing the Pt/C electrode by 0.25 V (Figure 13f,g). Researchers have devoted a lot of energy to the rapid development of photo‐assisted Zn‐air batteries, especially the emergence of the novel three‐electrode systems. We can synergistically accelerate the ORR and OER by combining the photoelectrode materials with different tendencies. The design principle of this photoelectrode material combination is clear: the CB position of the OR photoelectrode material is more negative, and the VB position of the OE photoelectrode material is more positive.

#### 3.2.2 Photo‐Assisted Zn‐Ion Battery

**Figure 14:**
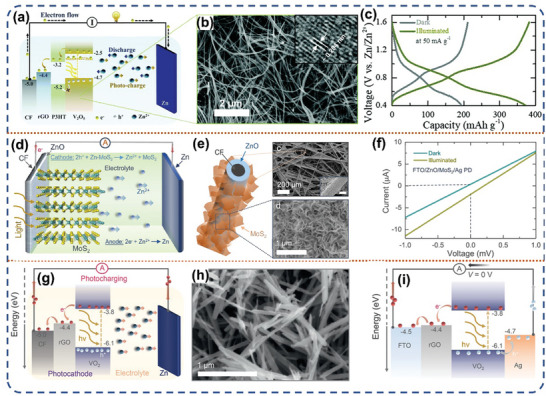
a) Schematic illustration of the photo‐charging mechanism of photo‐assisted Zn‐ion Battery. b) SEM image of V_2_O_5_ nanofibers and inset showing a high‐resolution TEM image. c) GCD profiles at 50 mA g^−1^ current density in dark and illuminated conditions (λ≈455 nm, intensity ≈12 mW cm^−2^). Reproduced with permission.^[^ ^124^ ^]^ Copyright 2020, Royal Society of Chemistry. d) Schematic illustration of the proposed photo‐charging mechanism of photo‐assisted Zn‐ion battery. e) Schematic illustration of MoS_2_ nanosheets grown on a ZnO coated carbon fiber and the SEM images of the photocathode at low and high magnifications. f) *I*–*V* curves of a stacked FTO/ZnO/MoS_2_/Ag PD in dark and illuminated (λ≈455 nm) states. Reproduced with permission.^[^ ^125^ ^]^ Copyright 2021, American Chemical Society. g) Schematic illustration of the proposed photo‐charging mechanism of VO_2_‐rGO photo‐assisted Zn‐ion battery. h) SEM image of the as‐synthesized VO_2_ nanorods used in the photocathodes. i) Energy band diagram of the stacked PD design. Reproduced with permission.^[^ ^126^ ^]^ Copyright 2021, Wiley‐VCH.

Rechargeable zinc‐ion (Zn‐ion) batteries are regarded as highly appealing energy storage systems in the era following lithium due to their advantageous features such as safety, affordability, moderate energy density, and straightforward preparation process. Compared to the batteries utilizing other types of single‐charged ions (Li^+^, Na^+^, K^+^) and divalent metal ion batteries (Mg^2+^, Ca^2+^), Zn‐ion batteries have the following advantages: 1) The theoretical specific and volumetric capacity of zinc metal is significantly high, measuring 5855 mAh cm^−3^; 2) The redox potential of Zn metal is suitable (−0.763 V vs NHE), which gives Zn‐ion batteries the ability to operate stably in aqueous electrolytes, thereby providing the benefits of affordability, security and eco‐friendliness; 3) Zinc element is abundant, non‐toxic and inexpensive (2 USD kg^−1^).^[^ ^120^, ^121^, ^122^ ^]^ It has inspired researchers to focus on the use of photo‐involved Zn electrochemistry to enhance the electrochemical efficiency of Zn‐ion batteries.^[^ ^123^ ^]^ Volder et al. proposed a new photo‐charging cathode material containing a mixture of vanadium pentoxide (V_2_O_5_), polymer (3‐hexylthiophene‐2, 5‐diyl) (P3HT) and reduced graphene oxide (rGO).^[^ ^124^ ^]^ V_2_O_5_ exhibits a considerable reversible capacity of ≈375 mAh g^−1^ and possesses an appropriate bandgap that enables the absorption of visible light (≈2.2 eV). This well‐designed composite has a regular arrangement of energy band structure, which can guide photoelectrons on the CB of V_2_O_5_ sequentially to the carbon fiber (CF) via P3HT and rGO in the light irradiation chamber, effectively promoting the separation of photo‐generated electron‐hole pairs (**Figure** **14**a,b). Based on this, the discharge capacity of the photo‐assisted Zn‐ion battery under irradiation reaches ≈370 mAh g^−1^, which is close to its theoretical limit (Figure 14c). They also successfully assembled a ≈64 cm^−2^ photo‐assisted Zn‐ion pouch cell with an optical window. Although photo‐assisted Zn‐ion batteries show remarkable potential for applications, the mechanically mixed composite cathode material requires additional conductive agents and binders, which can lead to terrible photo‐generated carrier separation and limited overall photo‐charging conversion efficiencies. To overcome this shortcoming, a new photoanode material has been reported by Volder et al.^[^ ^125^ ^]^ They directly grew ZnO film on the carbon filet (CF) as an electron transport and hole blocking layer, and then deposited the light trapping material MoS_2_ (Figure 14d,e). MoS_2_ has a narrower bandgap than V_2_O_5_ (1.9 vs 2.2 eV) and is able to better absorb visible light and excite more photoelectrons. The presence of photocurrent in the *I*–*V* curve in the event of no external voltage being present (*V *= 0 V) proves the effective photoconversion capability of the photoanode material (Figure 14f). This photo‐assisted Zn‐ion battery can be charged directly by solar without an external power supply, and the capacity was increased from 245 to 340 mAh g^−1^ at a current density of 100 mA g^−1^. They also used VO_2_@rGO as the photocathode for the photo‐assisted Zn‐ion battery(Figure 14g,h).^[^ ^126^ ^]^ Surprisingly, this photocathode material exhibits long cycling stability: ≈90% of the capacity is retained after 1000 cycles. At the same time, a specific capacity of up to 315 mAh g^−1^ was demonstrated at 200 mA g^−1^ (Figure 14i). It gives photo‐assisted Zn‐ion batteries great potential as an innovative technology to address energy shortages.

#### 3.2.3 Other Types of Photo‐Assisted Zn‐Based Metal battery

**Figure 15:**
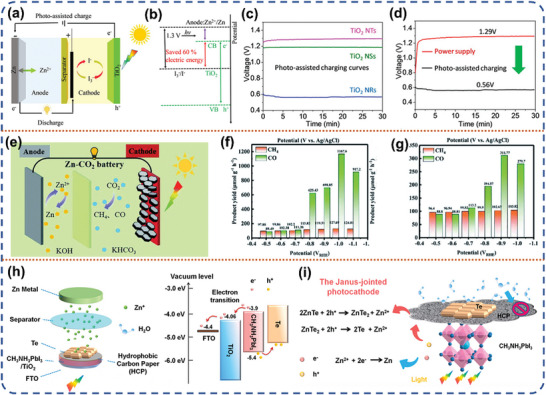
a) Schematic illustration of a photo‐assisted chargeable Zn‐I_2_ battery with a three‐electrode configuration. b) The energy diagram of the designed photo‐assisted Zn‐I_2_ battery. c) The charge curves of photo‐assisted chargeable aqueous Zn‐I_2_ battery at a current of 0.01 mA cm^−2^ containing TiO_2_ NTs, TiO_2_ NSs, and TiO_2_ NRs as the photoelectrode, respectively. d) The charge curves of the Zn‐I_2_ battery using TiO_2_ NRs as the photoelectrode (black lines for power supply charge and the red lines for photo‐assisted charge). Reproduced with permission.^[^ ^127^ ^]^ Copyright 2019, Wiley‐VCH. e) Schematic mechanism of photo‐assisted aqueous Zn‐CO_2_ battery. f) Photo‐electrocatalytic CO_2_ reduction reaction product yields at different potentials of U‐Cu_2_O/CuCoCr‐LDHs. g) Electrocatalytic CO_2_ reduction reaction product yields at different potentials of U‐Cu_2_O/CuCoCr‐LDHs. Reproduced with permission.^[^ ^128^ ^]^ Copyright 2021, Royal Society of Chemistry. h) Schematic representation of the integrated photo‐rechargeable aqueous Zn‐Te battery and corresponding energy band diagram. i) Dual‐functional photoelectrode for the direct photo‐charging process. Reproduced with permission.^[^ ^129^ ^]^ Copyright 2023, American Chemical Society.

In 2019, Li et al. introduced the concept of photo‐assisted Zn‐iodine (Zn‐I_2_) batteries.^[^ ^127^ ^]^ They used an I_3_^−^/I^−^ dyad and a TiO_2_ semiconductor as the anode and photocathode, respectively, to achieve the process of converting solar energy into electrical energy in the Zn‐ I_2_ battery system (**Figure** **15**a,b). During the photo‐charging process, photoelectrons are transferred to the anode to reduce Zn^2+^ to Zn, and photo‐generated holes oxidize the I_3_^−^ to I^−^. They regulating the TiO_2_ with different morphology, which are TiO_2_ nanospheres (TiO_2_ NSs), TiO_2_ nanorods (TiO_2_ NRs) and TiO_2_ nanotubes (TiO_2_ NTs). It was found that TiO_2_ NRs photoelectrode can reduce the charging voltage to 0.56 V, which is below the discharge voltage (1.2 V) and save 54% of external power supply (Figure 15c,d). Aqueous Zn‐CO_2_ rechargeable batteries have garnered significant interest owing to their distinct attribute of being able to immobilize CO_2_ and generate electricity. In 2021, Lu et al. used a p‐n heterojunction of Cu_2_O/CuCoCr‐LDH as the photocathode of a photo‐assisted Zn‐CO_2_ battery.^[^ ^128^ ^]^ The efficient separation of photo‐generated carriers driven by the built‐in electric field not only effectively accelerates the electrocatalytic conversion of CO_2_ to the discharge products CO and CH_4_, but also accelerates the water oxidation reaction (Figure 15e). At −1.0 V versus RHE, the CO yield can reach 1167.6 umol g^−1^ h^−1^, which is four times higher than that in the absence of light, as well as up to 90.14% of CO selectivity (Figure 15f,g). Meanwhile, the round‐trip efficiency of the photo‐assisted device reaches 58.94%, which is almost three times higher than in the dark state. It provides a new way for CO_2_ consumption and power generation.

Latest, Li et al. reported the customized design of a photocathode with a Janus joint structure, which combines perovskite materials and tellurium composite electrodes to achieve high efficiency. This innovative photocathode is then applied in an aqueous zinc‐tellurium (Zn‐Te) battery (Figure 15h)^[^ ^129^ ^]^ The photoelectrode with a well‐matched energy level structure ensures efficient transfer of photo‐generated charges and their conversion into electrical energy (Figure 15i). As expected, this Te/CH_3_NH_3_PbI_3_/TiO_2_ photocathode showed a charging voltage drop of 0.1 V and an additional capacity of 362 mAh g^−1^ under light. At a current density of 1000 mA g^−1^, the prepared photocathode showed an 83% increase in specific capacity. The overall efficiency achieved 12%, while the PCE reached 0.31%, which is in line with the performance of typical photo‐rechargeable batteries. Impressively, the current generated by the perovskite (CH_3_NH_3_PbI_3_) under light can directly charge the battery without external current, which demonstrates the photo‐charging behavior of the perovskite and the self‐powered application characteristics. This structure enhances the separation of photo‐generated carriers and expedites the redox kinetics. These findings suggest that the integrated Te/CH_3_NH_3_PbI_3_/TiO_2_ composites provide a sustainable mode of energy harvesting and storage as a photocathode in the direct photo‐charged aqueous systems.

### 3.3 Other Photo‐Assisted Metal‐Based Battery

**Figure 16:**
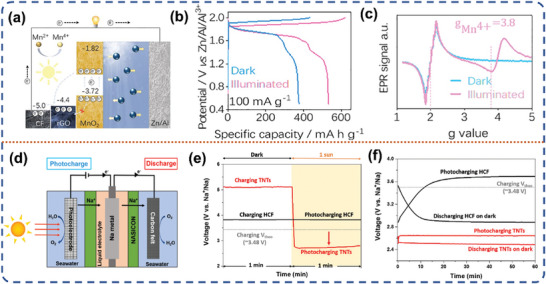
a) The separation process of electron‐hole pairs based on energy level matching. b) Galvanostatic charge/discharge curves carried out with/without illumination at 100 mA g^−1^. c) Corresponding g value distribution. Reproduced with permission.^[^ ^130^ ^]^ Copyright 2021, Elsevier B.V. d) Schematic illustration of the solar seawater battery, in which the information is divided into the photo‐charge part and discharge part. e) Galvanostatic charging (dark and 1 Sun irradiation) at 0.015 mA cm^−2^ for the TNTs photoanode and HCF cathode. f) Initial charge and discharge curves for the TNTs photoanode and HCF cathode at 0.015 mA cm^−2^. Reproduced with permission.^[^ ^11^ ^]^ Copyright 2019, Elsevier Ltd.

Aluminum (Al) is the most abundant metal element in the earth's crust, accounting for ≈8% of the total. Al element is an excellent candidate for the ion battery, owing to its low cost, abundant resources and high energy density. That is why the basic research of Al‐ion batteries is in full swing. Limited by the slow kinetics and undesired diffusion of soluble Mn^2+^, the electrochemical performance of MnO_2_ based Al‐ion battery is difficult to fully unleash. Based on the n‐type semiconductor characteristics of the MnO_2_ cathode and its property to be excited by visible light, Jiao et al. introduced the photo‐generated carriers inside the Al‐MnO_2_ battery system to achieve accelerated dynamics, fast charging and enhanced rate performance.^[^ ^130^ ^]^ Through a reasonable energy level structure sequence, the efficient separation of photo‐generated electron‐hole pairs are accelerated and the photo‐charging effect of photoelectrons is fully utilized (**Figure** **16**a). The MnO_2_ cathode releases a high discharge capacity of 531 mAh g^−1^ under light irradiation, representing a 41.3% increase in capacity compared to the dark state (Figure 16b). At the same time, photo‐generated holes can oxidize the soluble Mn^2+^ into Mn^4+^ during the discharging process, effectively improving the utilization of active materials (Figure 16c). In addition, choi et al. used TiO_2_ nanotube arrays (TNTs) as photoelectrode and Na metal as the anode to construct an effective photoelectrochemical‐assisted rechargeable seawater battery (PARSB).^[^ ^11^ ^]^ The photo‐generated holes generated by TNTs are used to drive the oxygen evolution reaction, effectively reducing the charging voltage of PARSB to ≈2.65 V (Figure 16d,e). In contrast, the photo‐charge voltage and discharge voltage of the heated carbon felt cathode are ≈3.8 and ≈2.9 V respectively (Figure 16f). It is foreseeable that Al and Na metals are cheap and suitable battery materials. By pairing them with proper photoelectrode materials and optimizing the battery configurations, photo‐assisted rechargeable metal batteries based on Al/Na metal also have broad prospects.

## 4 Summary and Perspectives

**Figure 17:**
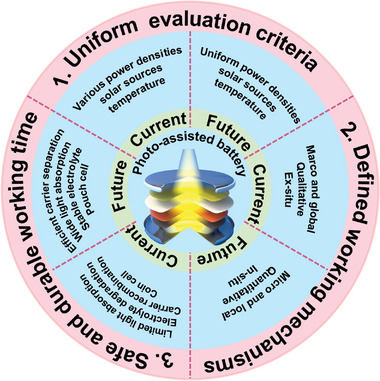
Current challenges and future perspectives of photo‐assisted rechargeable metal batteries.

In this review, we first introduce the key battery concepts, photoelectrochemical concepts and photo‐assisted battery concepts, respectively. The battery concept section focuses on the intercalation electrochemistry and conversion electrochemical reaction mechanisms. The concept of photo‐electrochemistry focuses on the process of converting solar energy into electrical energy and how photosensitive materials participate in the redox reactions. Subsequently, the specific role of the integrated photoelectrode in the photo‐assisted battery system and the cyclic conversion between solar energy and electrical energy are explained in detail by combining the mechanism diagram and text description. Finally, we also provide a comprehensive overview of the structural design and mechanism of photoelectrode materials participate in various three‐electrode and two‐electrode systems, accompanied with specific examples. Despite the significant progress achieved in recent years with photo‐assisted rechargeable metal batteries, including lower polarization voltages, longer cycling life, higher round‐trip efficiencies, and expand capacity release, there are still several challenges that require further research (**Figure** **17**).

First of all, standardized performance evaluation and unified test conditions should be established to enable more reasonable and effective assessment of the performance of photo‐assisted rechargeable batteries, as well as to determine the depth of progress and the feasibility of moving toward practical application. In the existing literature on photo‐assisted device, the range of wavelengths of simulated sunlight used varies in power intensity, making it difficult to compare them reasonably. It is evident that excess electrolyte and high energy light favor battery performance. Accurate decoupling of the photo‐thermal and photo‐electric effects is essential for a fair and reasonable comparison of the performance of photo‐assisted devices.^[^ ^131^ ^]^

Second, the working mechanism of photo‐assisted rechargeable batteries remains to be elucidated. Therefore, more reasonable verification experiments need to be designed and combined with density functional theory (DFT) and effective in situ characterization techniques to synergistically clarify the effective principle of the photo‐assisted system. During the charging and discharging process, rechargeable metal batteries experience a series of electrochemical reactions. Including ion transport, adsorption‐activation of reactive species and multi‐electron redox reactions. The introduction of light irradiation further complicates these reactions. Current research has focused on developing new catalysts and structural designs. However, it lacks an in‐depth exploration of the essential photochemical and electrochemical processes involved in charging and discharging process. It is important to provide a clear and comprehensive understanding of these processes to advance the field. There are often overlooked details specifically related to the failure of photo‐assisted batteries. Does it stem from photo‐corrosion of the electrolyte/photocatalyst? Or is it the accumulation of side‐products from the cyclic catalytic process? Or is it the loss of active material and failure of the anode metal by pulverization? Therefore, in situ characterization methods, commonly used in traditional metal‐based batteries, can effectively aid in exploring the working mechanism of photo‐assisted rechargeable metal batteries. For instance, the ISI‐XPS is widely used in photocatalysis field to directly observe the transport paths of photo‐generated carriers and the active sites of photocatalyst.^[^ ^29^ ^]^ It has the potential to clarify the pathways of redox reactions and identify the catalytic active sites in the photo‐assisted battery system. Other techniques such as in situ transmission electron microscopy and in situ Raman spectroscopy can directly observe the evolution of structure and morphology of charging and discharging products. When combined with density functional theory and other theoretical analysis methods, these techniques can synergistically verify the adsorption and mass transfer effects of active substances and intermediates in the redox reaction.

Third, the development of photo‐assisted rechargeable metal batteries is still in the early stages. Therefore, the focus should be on solving the safety and stability issues of photo‐assisted rechargeable devices. Only after the excellent performance of the coin‐type photo‐assisted rechargeable battery is promoted to the pouch cell, can we pave the way for the practicalization of photo‐assisted energy storage devices. Many current devices have a short cycle life and poor rate performance due to the limited light trapping, severe recombination of carriers and electrolyte decomposition or side reactions. The inefficient light‐absorption capacity and severe recombination of carriers lead to slight electrochemical performance enhancement but result in additional interfacial impedance between the photocatalysts and collectors. Reasonable design of the photocathode configuration can accelerate the separation of carriers and reduce the interfacial mass transfer resistance. Electrolyte decomposition and side reactions under the light irradiation can lead to the loss of electrolyte and active species and limit the cycle life of the device. Developing more stable gel electrolytes and solid‐state electrolytes would be an effective solution. Demonstrating the photo‐assisted rechargeable battery system at the pouch cell level as soon as possible is crucial to showcase the feasibility of commercial application of photo‐assisted energy storage devices in a timely and effective manner.

In conclusion, photo‐assisted rechargeable metal batteries present an innovative approach to fulfill the growing need for energy while tackling the issue of greenhouse gas emissions. It offers motivation for the advancement of novel eco‐friendly energy apparatus, including solar‐powered rechargeable automobiles. Existing challenges are present in various research areas, including photo‐electrochemistry, materials science, semiconductor physics and electronics. Hence, addressing these issues necessitates the adoption of a cross‐disciplinary methodology. With increasing efforts being made in the field of photo‐assisted energy storage devices, photo‐assisted rechargeable metal batteries are expected to become practical in the near future and change the way people travel and the ways of energy supply.

## Conflict of Interest

The authors declare no conflict of interest.
